# SenPred: a single-cell RNA sequencing-based machine learning pipeline to classify deeply senescent dermal fibroblast cells for the detection of an in vivo senescent cell burden

**DOI:** 10.1186/s13073-024-01418-0

**Published:** 2025-01-14

**Authors:** Bethany K. Hughes, Andrew Davis, Deborah Milligan, Ryan Wallis, Federica Mossa, Michael P. Philpott, Linda J. Wainwright, David A. Gunn, Cleo L. Bishop

**Affiliations:** 1 Blizard Institute, Barts and The London Faculty of Medicine and Dentistry, Queen Mary University of London, London, E1 2AT UK; 2 Unilever R&D, Merritt Blvd, Trumbull, CT 06611 USA; 3 Unilever R&D, Colworth Science Park, Sharnbrook, Bedfordshire, MK44 1LQ UK

**Keywords:** 3D organotypic culture, In vivo senescence detection, Living skin equivalent, Machine learning, scRNA-seq, Senescence

## Abstract

**Background:**

Senescence classification is an acknowledged challenge within the field, as markers are cell-type and context dependent. Currently, multiple morphological and immunofluorescence markers are required. However, emerging scRNA-seq datasets have enabled an increased understanding of senescent cell heterogeneity.

**Methods:**

Here we present SenPred, a machine-learning pipeline which identifies fibroblast senescence based on single-cell transcriptomics from fibroblasts grown in 2D and 3D.

**Results:**

Using scRNA-seq of both 2D and 3D deeply senescent fibroblasts, the model predicts intra-experimental fibroblast senescence to a high degree of accuracy (> 99% true positives). Applying SenPred to in vivo whole skin scRNA-seq datasets reveals that cells grown in 2D cannot accurately detect fibroblast senescence in vivo. Importantly, utilising scRNA-seq from 3D deeply senescent fibroblasts refines our ML model leading to improved detection of senescent cells in vivo*.* This is context specific, with the SenPred pipeline proving effective when detecting senescent human dermal fibroblasts in vivo, but not the senescence of lung fibroblasts or whole skin.

**Conclusions:**

We position this as a proof-of-concept study based on currently available scRNA-seq datasets, with the intention to build a holistic model to detect multiple senescent triggers using future emerging datasets. The development of SenPred has allowed for the detection of an in vivo senescent fibroblast burden in human skin, which could have broader implications for the treatment of age-related morbidities.

All code for the SenPred pipeline is available at the following URL: https://github.com/bethk-h/SenPred_HDF.

**Supplementary Information:**

The online version contains supplementary material available at 10.1186/s13073-024-01418-0.

## Background

Senescence is a fundamental cellular programme defined as a stable cell cycle arrest. The lack of universal senescence markers is a well-recognised challenge within the field. Hallmarks of senescence are both cell-type and trigger dependent, and no single marker alone is sufficient to confirm senescence in all contexts [1, 2]. It is, therefore, necessary to assess multiple markers encompassing cellular morphology, effectors of cell cycle arrest, the senescence-associated secretory phenotype (SASP), mitochondrial changes, chromatin remodelling, and lysosomal markers such as senescence-associated beta-galactosidase (SA-β-Gal) [1–3]. However, choosing appropriate markers for a particular experiment often relies on either literature precedent using the specific cell type and trigger, or lengthy optimisation and marker validation. Recent work has sought to address this by focusing on the development of accessible, stepwise, and standardised workflows for the classification and subsequent characterisation of senescence [2, 4]. Kohli et al. proposed a two-step approach to senescence detection, firstly focusing on more general lysosomal changes (SA-β-Gal) and cell cycle arrest (lack of EdU incorporation, and immunocytochemistry for Ki67, p16 and p21), and secondly on subtype-specific proinflammatory SASP changes [4].

This seminal work has allowed for a more systematic and streamlined approach to senescence detection. The outcome of these approaches continues to depend upon the senescence context, meaning prior knowledge of the subtype is required to determine a senescent-cell state. This renders in vivo senescent cell detection a current challenge, due to a lack of ‘ground truth’. Further, using a small number of selected markers could bias senescent cell detection towards a specific subtype and overlook additional heterogeneous subpopulations. Therefore, understanding the nuanced changes between senescence subtypes could allow for context-specific senescence detection and would enable unbiased classification of datasets where the senescent cell status is unknown. Furthermore, this understanding would remove the likelihood of detecting false positive and false negative data, which is crucial for progression within the senescence field. With the emergence of high-throughput screening methodologies, and ‘omics’-based sequencing technologies, our understanding of senescent cell subtypes is rapidly expanding. This necessitates the development of tools which can utilise this data to classify senescence in novel datasets.

Machine learning (ML) methodologies are widely used to classify distinct cellular states [5]. The data must have a state of ground truth, so prior knowledge of the input class is required. The data can then be divided into training and testing datasets. The training dataset can be used to build the model, and the unseen testing dataset can be used to evaluate model performance based on a variety of metrics such as classification accuracy [6]. These ML models can then be applied to classify novel datasets. ML has been successfully applied to senescence classification in a small number of contexts (reviewed in Hughes, Wallis & Bishop 2023 [5]). For example, Heckenbach et al. have developed their model ‘Xception’ using neural networks, which can accurately classify a cell as senescent based on its nuclear morphology [7]. Alongside morphology, RNA sequencing data has also been used to build predictive models based on mRNA expression levels of senescent versus proliferative cells [8–11]. Jochems et al. used an elastic net model to accurately classify therapy-induced senescent cancer cells grown in a 2D monolayer [11]. Importantly, the group stratified the cells into those which had undergone short- and long-term treatments and found that treatment time strongly influenced the transcriptome of the cells. When applying their model to in vivo patient-derived xenografts treated with the senescence inducer SHP099, all xenografts were classified as ‘not senescent’. However, the group described that the use of bulk RNA sequencing datasets could explain why they were unable to detect senescence signatures in vivo.

Therefore, we asked if using single-cell transcriptomics alongside ML could improve senescent cell detection, particularly within heterogeneous in vivo contexts. Initially, we performed single-cell RNA sequencing of deeply senescent human dermal fibroblast (HDF) cells from a two-dimensional monolayer to build a ML model of senescence. We established that the model can accurately detect fibroblast senescence in independent 2D in vitro datasets, and in line with work by Jochems et al., we demonstrate that temporal kinetics is an important consideration when building transcriptomic senescent cell models. Interestingly, we find that fibroblasts grown in a two-dimensional monolayer cannot generate an accurate model of in vivo senescence. Therefore, to more faithfully replicate conditions in vivo*,* we performed single-cell RNA sequencing of cells grown in a three-dimensional matrix. It is widely reported that cells grown in 3D are more similar to cells in vivo, and can better recapitulate cellular morphologies, gene expression, and most notably the extracellular matrix [12–15]. We demonstrate that the 3D ML model enabled the detection of senescent dermal fibroblasts in vivo. Here, we present SenPred, an exploratory proof-of-concept pipeline, which lays the foundation for a new approach to detect senescent cells in vivo and could allow for scRNA-seq-based prediction of alternative disease states.

## Methods

### Cells and reagents

Primary human dermal fibroblasts (HDFs) were a kind donation from anonymous healthy patients under standard ethical practice, reference LREC No. 09/HO704/69. HDFs were cultured in DMEM with 4 mM L-glutamine (41,966–029, Life Technologies) supplemented with 10% foetal bovine serum (1001/500, Biosera), in the absence of antibiotics, and maintained at 37 °C, 5% CO_2_, 95% humidity. Cells were seeded at 4000 cells/cm^2^ for passages 1–19, 7500 cells/cm^2^ for passage 20–25, and 10,000 cells/cm^2^ for passage 26 onwards. HDFs were serially passaged and were classified as early proliferative (EP) up to passage 13, and Deeply Senescent (DS) beyond passage 39 + 3. All cells were routinely tested for mycoplasma and were shown to be negative.

Dermal gels were generated by suspending HDFs in collagen 1 (354,236, Corning) supplemented with Matrigel (7,341,100, Corning), FCS (1001/500, Biosera), 10X DMEM (11,430–030, Invitrogen), and pH balanced with 4 M NaOH in the absence of antibiotics. HDF-gel mix was added dropwise to transwells (CC405, Corning) to give a final cell density of 10,000 HDFs per gel. Nine gels were constructed per condition. Dermal gels were maintained at 37 °C, 5% CO_2_, and 95% humidity.

### Single-cell RNA sequencing

For 2D samples, cells were grown in 6-well plates at 7000/cm^2^ for 72 h. Cells were collected, and resuspended in HBSS (14,025,092, Thermofisher), and immediately sorted into single cell oil droplet suspension. To extract the cells from 3D dermal gels, gels were incubated with collagenase D (Sigma) at 37 °C for 1 h. Collagenase activity was subsequently inhibited by HBSS addition. Gels were strained and centrifuged to capture cell pellets, which were resuspended in HBSS before sorting into single-cell oil droplet suspension.

Library generation and sequencing were performed using the v2 Chromium Single Cell 3′ Kit (10X genomics). Samples were sequenced using the Illumina NextSeq 500 High Output Run Sequencing platform using paired-end sequencing and aligned to the human genome (GRCh37) using Cell Ranger (10 × Genomics).

### External data acquisition

For external model testing, data was acquired from the published papers listed in Table 1.

**Table 1:** External published datasets used with SenPred

Reference	Accession	Sample	Context
Chan et al. [16]	GEO #GSE175533 [17]	WI-38 replicative senescent cells	In vitro
Teo et al. [10]	GEO #GSE115301 [18]	OIS and paracrine ER:IMR90 fibroblasts	In vitro
Tabib et al. [19]	The pre-processed dataset, including the raw UMI data matrix and associated metadata from Tabib et al., was acquired from the corresponding author, Robert Lafyatis (lafyatis@pitt.edu) in 2018	Whole skin	In vivo
Solé-Boldo et al. [20]	GEO #GSE130973 [21]	Whole skin	In vivo
Ganier et al. [22]	ArrayExpress: “E -MTAB- 13085” [23]	Whole skin	In vivo
Sikkema et al. [24]	Integrated human lung cell atlas: https://cellxgene.cziscience.com/collections/6f6d381a-7701–4781-935c-db10d30de293. [25].	Whole lung	In vivo

The datasets from Chan et al. and Teo et al. were used to apply and test the 2D SenPred pipeline on in vitro scRNA-seq experiments, whereas the datasets from Tabib et al., Solé-Boldo et al., Ganier et al., and Sikkema et al. were used to test the 3D SenPred pipeline. The number of cells analysed for the in vitro studies was as follows:

- Chan et al. [16]: PDL25, 1225; PDL29, 1619; PDL33, 1702; PDL37, 1260; PDL46, 2105; PDL50, 1042.
- Teo et al. [10]: Growing, 207; OIS, 240; Paracrine, 33.

The number of samples used for each in vivo study after QC and any additional sample details are listed in Tables 2, 3, 4, and 5. The ethics approval and data acquisition of human tissue were acquired by the respective authors within the in vivo references listed in Table 1.

**Table 2:** Donor metadata and cell counts following QC, for whole skin scRNA-seq by Tabib et al. [19]

Donor age	Sex	Sample site	Total number of cells	Number of fibroblast cells
23	Female	Dorsal mid-forearm	1185	648
24	Male	Dorsal mid-forearm	861	43
54	Male	Dorsal mid-forearm	1055	268
62	Female	Dorsal mid-forearm	1347	808
63	Male	Dorsal mid-forearm	791	253
66	Female	Dorsal mid-forearm	1659	538

**Table 3:** Donor metadata and cell counts following QC, for whole skin scRNA-seq by Solé-Boldo et al. [20]

Donor age	Sex	Sample site	Total number of cells	Number of fibroblast cells
25	Male	Inguinoiliac region	2769	666
27	Male	Inguinoiliac region	2395	813
53	Male	Inguinoiliac region	3320	1188
69	Male	Inguinoiliac region	4531	2034
70	Male	Inguinoiliac region	2139	592

**Table 4:** Donor metadata and cell counts following QC, for whole skin scRNA-seq by Ganier et al. [22]

Donor age	Sex	Sample site	Total number of cells	Number of fibroblast cells
56	Male	Nose	12579	5114
59	Male	Temple	11751	2597
62	Male	Cheek	2335	617
70	Male	Forehead	6209	1805
73	Male	Forehead	7953	620
77	Male	Ear	9205	1914
78	Male	Temple	4740	2184
80	Male	Forehead	12,119	1266
81	Female	Cheek	7439	2062
85	Male	Forehead	13,454	3153
86	Male	Ear	6982	2166
90	Male	Cheek	13,784	2503

**Table 5:** Donor metadata and cell counts following QC, for whole lung scRNA-seq by Sikkema et al. [24]

Donor age or median of age group	Sex	Sample site	Total number of cells	Number of fibroblast cells
25	Male	Lung	49,574	3606
29	Male	Lung	17,533	361
30	Male + female	Lung	35,652	301
32.5	Female	Lung	3771	211
34	Female	Lung	9134	173
35	Male + female	Lung	17,711	173
42.5	Male	Lung	16,899	357
49	Male	Lung	14,613	125
50	Female	Lung	16,187	146
52	Male	Lung	41,505	3889
52.5	Male	Lung	2425	139
55	Male + female	Lung	25,789	346
57	Male	Lung	33,202	221
57.5	Male	Lung	7971	1358
59	Male	Lung	23,027	347
62	Male	Lung	7286	339
64	Male + female	Lung	16,258	263
67.5	Female	Lung	14,728	718

Additional preprocessing steps were carried out on both the Tabib et al. [19] and Solé-Boldo et al. [20] datasets, to combine donors and generate a single Seurat Object for each. Cell level filtering and normalisation of raw data from Tabib et al. [19] and Solé-Boldo et al. [20] were performed using Seurat R package version 3.2.2 in R version 4.0.3 [26]. To remove low-quality cells, the data was filtered using the following metrics:

- nCount_RNA > 500
- nFeature_RNA > 250
- nFeature_RNA < 7500
- percent.mt ≤ 5
- log10genes_per_UMI < 0.8

Data was normalised using ScTransform [27], and the sctransform normalized counts for each sample were then integrated [26], using the top 3000 most variable genes in the SelectIntegrationFeatures() function. Then, the FindIntegrationAnchors() function was applied with default parameters to identify the anchors across the different samples in each dataset. The anchors were then passed to the IntegrateData() function to create a single normalized and integrated Seurat object for each dataset.

### Cell-level filtering and normalisation

All subsequent data analysis was performed using R (version 4.2.2) in Rstudio (2022.07.2 Build 576), running under macOS Monterey 12.0.1. Matrix, gene, and barcode.gz data files were loaded and converted into a Seurat object [28]. Unless otherwise stated, default parameters from Seurat V4.3.0.1 were applied. To remove low-quality cells, the data was filtered using the following metrics (Supplementary Fig. S1):

- nCount_RNA > 1000
- nFeature_RNA > 400
- percent.mt ≤ 12

Data was normalised using NormalizeData(), which normalises individual gene expression of a cell to total gene expression of this cell and multiplies by the default scale factor (10,000). This data is then log transformed.

To subset fibroblasts from whole skin data for the Tabib [19] and Solé-Boldo [20] datasets, pre-defined metadata was used. For Ganier et al. [22], dot plots were generated to visualise the mean cluster expression of a pre-defined list of genes from the original paper, using the Seurat function DotPlot(). To identify lung fibroblasts in Sikkema et al., data was subset for pre-defined fibroblasts, from healthy donors, where the age was reported. Donors were removed if they had less than 100 fibroblasts recorded.

### Dimensionality reduction and differential gene expression analysis

The top 2000 variable genes were identified using the FindVariableFeatures function [26], and these were subsequently scaled to ensure a mean expression of 0 and variance of 1 across all cells. K-nearest neighbour algorithms were used based on PCA distance, followed by the Louvain algorithm to determine clusters. The resolution of all cluster-based functions was determined systematically using the Clustree package [29]. These clusters were visualised on UMAP plots. To identify differentially expressed genes (DEGs) in the clusters, FindAllMarkers default Seurat function was implemented. Genes must be detected in > 25% of cells in at least 1 cluster and have a logFC greater than 0.25.

### Machine learning senescence classifier

The ScPred package developed by the Powell group was used to build ML models to classify senescence (https://github.com/powellgenomicslab/scPred/) [30]. Unless otherwise stated, default parameters of ScPred V1.9.2 were used. Data was separated into training (80%) and testing (20%) datasets. ScPred ML models were then built on training data using all principal components, using a variety of methods including Support Vector Machines with Radial Basis Function Kernel (SVM), Mixture Discriminant Analysis (MDA), K-nearest neighbours (KNN), and Generalised Linear Model (GLM). The models were evaluated based on their classification of the testing dataset through a variety of methods, including ROC, sensitivity, and specificity, and the best performing model (MDA) was taken forwards. This utilised the CRAN package mda_0.5–4. For the MDA model, a fivefold cross-validation was used. These models could then be applied to classify senescence in alternative datasets.

### Trajectory analysis

Monocle3 V1.3.1 was used to analyse the trajectory of the PDL50 cells [31–33]. All default parameters were used unless otherwise stated. The earliest principal node was calculated using the function get_earliest_principal_node, where the node most surrounded by EP cells was selected. The cells were then ordered based on pseudotime.

### Matrisome analysis

NABA_MATRISOME human gene set was obtained from Petrov et al., and the median log normalised gene count expression was calculated for the 2D, 3D, Tabib et al. [19] and Solé-Boldo et al. [20] datasets. Genes which had a median count of zero for all conditions were removed, and the final list of 147 matrisome genes is reported in Supplementary Table S2. The gene counts were converted into a heatmap using heatmap.2(), and Ward hierarchical clustering was applied.

### Marker correlation plots

Normalised gene counts were extracted from the Tabib and Solé-Boldo data, using a list of pre-defined senescence genes: CDKN1A, CDKN2A, IGFBP3, SERPINE1, IGF1, CXCL12, IL6, MMP3 and TP53. The Pearson correlation coefficient was calculated between all pairs of genes, and Ward hierarchical clustering was applied to generate both x- and *y*-axis dendrograms. The results were then visualised on a heatmap using heatmap.2().

### Digital PCR

Early Proliferative (EP – P10 − P12) and Deeply Senescent (DS – P39 + 3) FLF1 HDF cell pellets were lysed with QIAzol (217,004, Qiagen miRNeasy Mini Kit), periodically vortexed for 5 min and snap frozen at -80C. Total RNA extraction was performed using the miRNeasy Mini Kit (217,004, Qiagen), following the manufacturer’s guidelines (“Quick-Start protocol miRNeasy Mini Kit” guide, Qiagen). Following this, RNA ethanol precipitation was performed to increase sample purity, where 30 mL of RNA was mixed with 75 μl of 100% ethanol (34,852, Sigma Aldrich), 3 μl of sodium acetate 3 M at pH 5.2 (R1181, Thermofisher Scientific), and 1 μl GlycoBlueTM Coprecipitant (AM9515, Invitrogen), and incubated overnight at − 20 °C. The sample was centrifuged at 12,000* g* for 10 min at 4 °C, resuspended in 500 μl of 80% EtOH, and centrifuged a second time at 12,000* g* for 10 min at 4 °C. The pellet was dried for 2 h at RT and resuspended in 20 μl RNAse-free distilled water (217,004, Qiagen miRNeasy Mini Kit).

Precipitated RNA was converted to cDNA using the SuperScriptTM III Reverse Transcriptase kit, as per the manufacturer’s instructions (18,080,044, Invitrogen). cDNA concentration was measured using the Qubit 2.0 Fluorometer (Thermofisher Scientific), and absolute quantification of cDNA was carried out using SYBR Green I dye (S7563, Thermofisher Scientific) on the Applied Biosystems QuantStudio Absolute Q Digital PCR System. Digital PCR was carried out using the Absolute Q DNA digital PCR Master Mix (A52490, Thermofisher Scientific), with a QuantStudio Absolute Q MAP16 plate kit (A52865, Thermofisher Scientific), as per manufacturer’s instructions. For analysis, absolute cDNA counts were normalised to 1 ng/ml of cDNA loaded.

## Results

### Generating and evaluating machine learning models of replicative fibroblast senescence

**Fig. 1:**
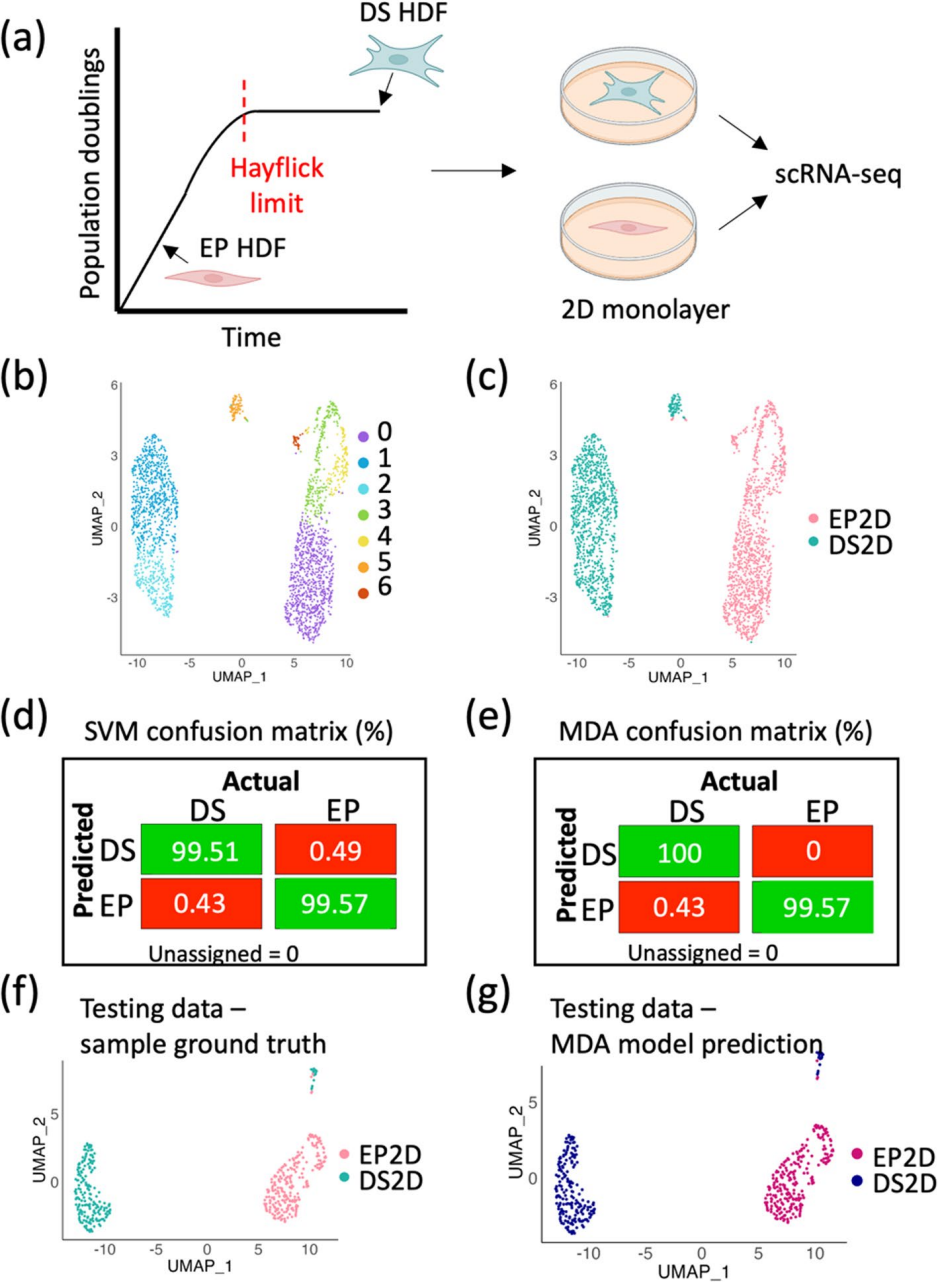
Machine learning models can accurately discriminate between early proliferative (EP) and deeply senescent (DS) human dermal fibroblasts. **a** Schematic illustration of the position of early proliferative (EP) and deeply senescent (DS) human dermal fibroblasts (HDFs) on the Hayflick curve. The samples were grown in a 2D monolayer before single-cell RNA sequencing (scRNA-seq). Created with BioRender.com. **b** Uniform manifold approximation projection (UMAP) of transcriptomic signatures of EP and DS fibroblasts. **c** UMAP in **b** overlayed with sample type. **d** Support vector machine (SVM) model confusion matrix generated from the testing dataset, highlighting true positives and true negatives (green), and false positives and false negatives (red). **e** Mixture discriminant analysis (MDA) model confusion matrix generated from the testing dataset, highlighting true positives and true negatives (green), and false positives and false negatives (red). **f** 20% testing dataset clustered into a UMAP and overlayed with sample type. **g** UMAP in **f** overlayed based on prediction using the MDA model, with the ScPred package

In an effort to develop a ML model of senescence classification, we cultured adult human dermal fibroblasts (HDFs) to replicative senescence (passage 39) and allowed them to reach a deeply senescent state over a further 3 weeks in culture (DS; P39 + 3). Single-cell RNA sequencing (scRNA-seq) was carried out on early proliferative (EP) and deeply senescent (DS) HDFs cultured in a two-dimensional monolayer (extensive model validation previously reported in Wallis et al. [34]) (Fig. 1a). Using *k*-nearest neighbours (KNN), seven independent clusters were identified in the EP and DS HDF dataset (Fig. 1b). Overlaying these clusters with the originating cell populations showed distinct clustering of EP and DS fibroblasts, highlighting the discrete mRNA profiles of the two populations, together with inherent heterogeneity within both the EP and DS fibroblast populations (Fig. 1c). Given that the EP and DS states were transcriptionally distinct, we proceeded to build a ML model to distinguish the two conditions.

The scRNA-seq data was split into training (80%) and testing (20%) datasets, for model development and evaluation. Four ML models were explored, namely support vector machine (SVM; Fig. 1d), mixture discriminant analysis (MDA; Fig. 1e), *k*-nearest neighbours (KNN) and generalised linear model (GLM) (Supplementary Table 1). The MDA confusion matrix was the most accurate and highlighted a marginally higher percentage of true positive DS cells than the SVM model (0.49% higher). Both the MDA and SVM models have an ROC score of 1, and a sensitivity of 0.999, but the MDA model had higher specificity than the SVM model (1 versus 0.99, respectively). KNN and GLM were also tested but had marginally lower evaluation metrics (KNN ROC: 0.998, Sens: 0.978, Spec: 0.992; and GLM ROC: 0.999, Sens: 0.996, Spec: 0.994, respectively). These evaluation metrics are displayed in Additional File 1: Table S1. For this reason, the MDA model was selected for subsequent work. Comparing the known classification of the testing dataset (Fig. 1f), with the MDA model predictions (Fig. 1g) shows a striking prediction accuracy of 99.79%. Although intra-experimental, this testing data is previously ‘unseen’, suggesting model overfitting is unlikely and instead, the two classification outcomes are transcriptionally distinct. This work demonstrates that it is possible to build an MDA ML model of senescence classification, which can accurately predict fibroblast deep senescence in a two-dimensional monolayer. Therefore, MDA machine learning models are used throughout this work.

### Testing the models on an external dataset reveals the importance of temporal senescence kinetics

**Fig. 2:**
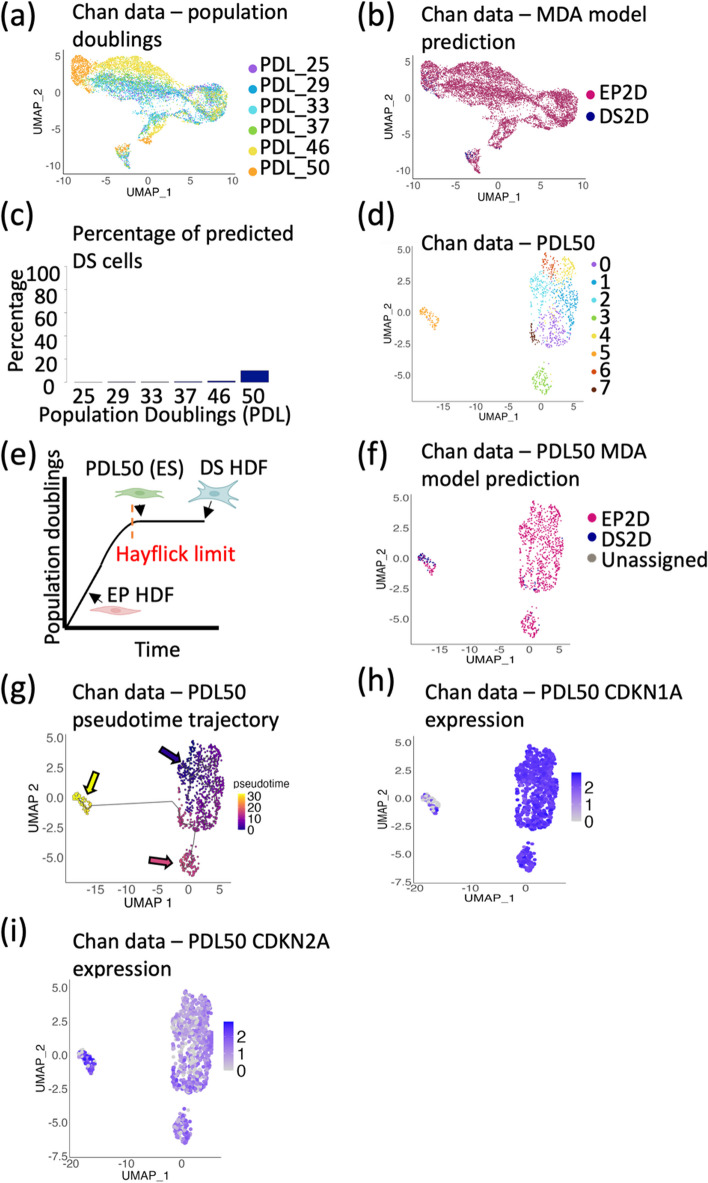
The machine learning model can detect deeply senescent but not early senescent fibroblasts. **a** UMAP of scRNA-seq data from fibroblasts from the external dataset (Chan et al. [16]) at different population doubling levels (PDL). **b** UMAP in **a** overlayed based on prediction from the MDA model. **c** Barplot representing the percentage of cells which are predicted to be deeply senescent (DS) at each PDL, using the MDA model. **d** UMAP of the PDL50 cells from Chan et al. [16]. **e** Schematic illustrating position on the Hayflick curve of early proliferative (EP) and deeply senescent (DS) human dermal fibroblasts (HDFs), and PDL50 or ‘early senescent’ cells. Created with BioRender.com. **f** UMAP in **d** overlayed based on prediction from the MDA model. **g** Monocle3 trajectory plot of UMAP in **d**, overlayed with predicted pseudotime trajectory. **h** PDL50 UMAP from **d**, overlayed with expression of CDKN1A. **i** PDL50 UMAP from **d**, overlayed with expression of CDKN2A

To test the MDA ML model’s performance, we used an independent publicly available dataset from Chan et al. (GEO—#GSE175533), which included scRNA-seq data for increasing population doubling levels (PDLs) of WI-38 fibroblasts [16]. These fibroblasts ranged from PDL25 to PDL50, the latter of which Chan et al. described as being in an early senescent state. The transcriptional differences of the PDL50 senescent cells were apparent when the cells were clustered and plotted onto a UMAP (Fig. 2a), with the majority of the PDL46 and PDL50 cells clustering away from the main bulk of the population. Applying our MDA ML model to the WI-38 fibroblast dataset shows an enrichment for DS predictions in the UMAP locations of the early senescent PDL50 cells (Fig. 2b). However, only 10.08% of the PDL50 cells are predicted to be DS (Fig. 2c). To further investigate this, the PDL50 cells were isolated and clustered independently, producing eight clusters (Fig. 2d). Intriguingly, cluster five was largely enriched for DS-predicted cells, suggesting that this subpopulation of cells was transcriptionally similar to the DS cells in the original HDF model.

We hypothesised that the low percentage of DS prediction in the PDL50 cells could be due to temporal differences in the two experiments. The DS cells used to build the MDA ML model had undergone three weeks of culture after reaching replicative senescence, to establish and deepen their phenotype. Conversely, the PDL50 WI-38 fibroblast cells from Chan et al. were sequenced immediately upon reaching their Hayflick limit (Fig. 2e). To test this hypothesis, we applied Monocle3 trajectory analysis to the PDL50 cells [33]. Cluster five (the cluster enriched for cells which were predicted to be DS—Fig. 2f), fell at the end of the predicted trajectory, suggesting that the model was detecting deeply senescent but not early senescent fibroblasts (Fig. 2g) within the PDL50 population.

Cells at PDL50 likely represent a heterogeneous population, where some cells have reached the Hayflick limit earlier and deepened their senescent phenotype. To confirm this, we examined the expression of known cell cycle arrest markers CDKN1A (p21) and CDKN2A (p16/Arf). Alcorta et al. reported an initial increase of p21 during the early stages of cell cycle arrest of human diploid fibroblasts, with a gradual increase of p16 expression as the senescence phenotype deepens [35]. Intriguingly, this was also apparent in the PDL50 cells, with those cells predicted to be DS having lower expression levels of CDKN1A (Fig. 2h) and higher expression levels of CDKN2A mRNA (Fig. 2i) than the remaining PDL50 population. It must be noted that an increase of CDKN2A expression could be due to increased expression of either p16 or Arf alternative reading frames. However, using sequence-specific primers, the DS HDFs yielded a significant increase in p16 mRNA and a significant decrease in Arf compared to their proliferative counterparts with senescence (Additional File 1: Fig. S2). It is therefore likely that there is an increased expression of p16 but not Arf mRNA in the PDL50 DS predicted cells. This increase supports our hypothesis that the MDA ML model is able to detect deeply senescent but not early senescent fibroblasts.

**Fig. 3:**
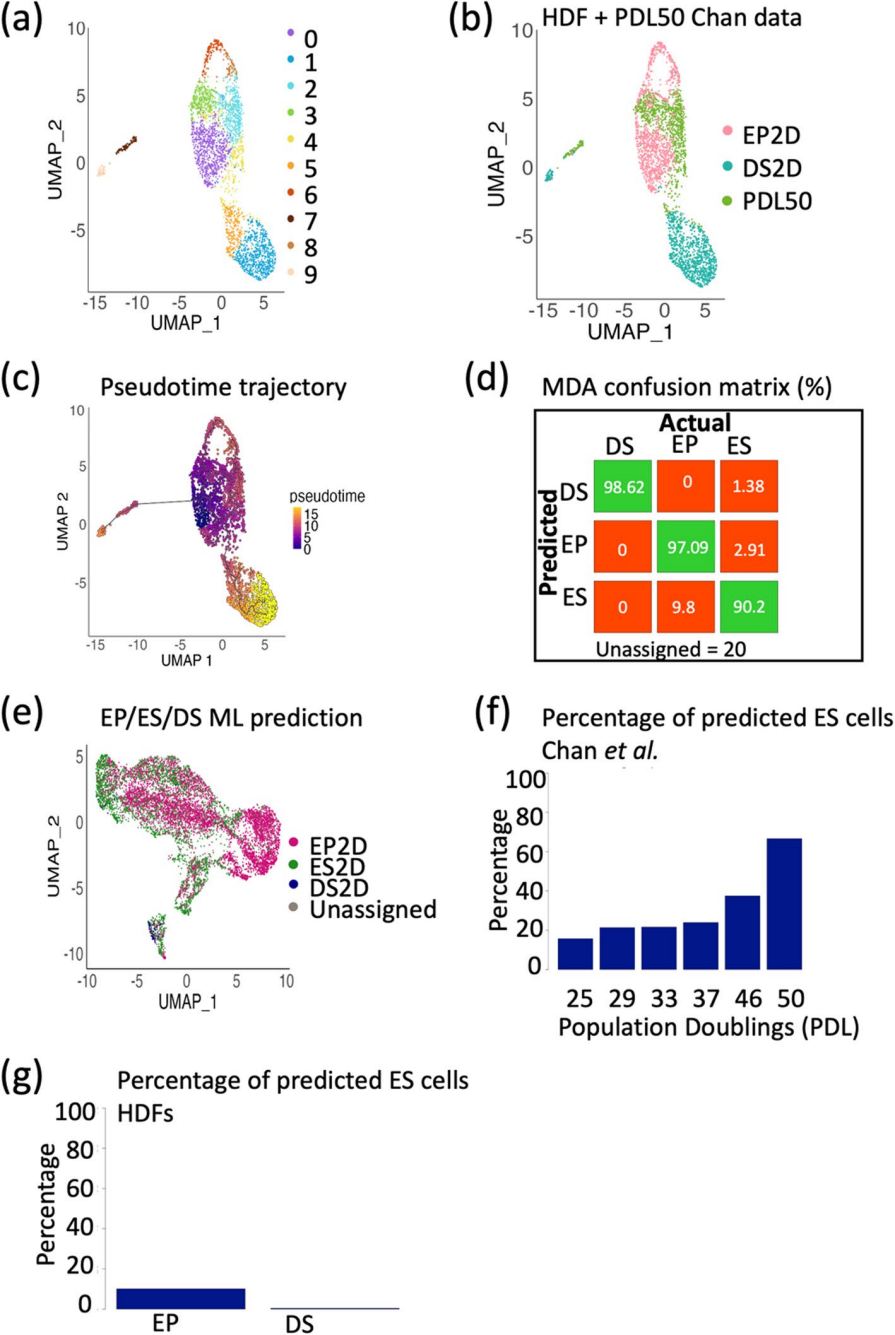
Building a machine learning model incorporating early senescent (ES) cells highlights high levels of heterogeneity throughout progressive population doublings. **a** UMAP of combined scRNA-seq data from EP and DS HDFs, and PDL50 WI-38 fibroblasts from Chan et al. [16]*.***b** UMAP in **a** overlayed with sample type. **c** Pseudotime trajectory of UMAP in **b**. **d** Confusion matrix of MDA model of EP, ES, and DS cells, highlighting true positives and true negatives (green), and false positives and false negatives (red). **e** UMAP of all WI-38 PDLs from Chan et al. [16] (Fig. 2a), overlayed with prediction from EP/ES/DS 2D model. **f** Barplot showing the percentage of predicted ES cells at each PDL. **g** Barplot showing the percentage of predicted ES cells within the EP and DS HDF populations

To build a ML model which could encompass early replicative senescent fibroblasts (ES), the PDL50 cells from Chan et al. [16] were incorporated into the model as an additional ES classifier. First, clustering the ES, EP, and DS cells revealed a more successive pattern, with the ES cells bridging the gap between the EP and DS cells (Fig. 3a–b). This was confirmed using Monocle3 trajectory analysis (Fig. 3c). This is perhaps unsurprising, as we have previously identified the ES cells to contain a heterogeneous mix with a small proportion of DS cells. The MDA confusion matrix highlights more than 90.2% true positives for all intra-experimental predictions, with the lowest percentage of true positives being for the ‘early senescent’ or ES cells (Fig. 3d), which could again be explained by the heterogeneity of this population.

Applying this model, the WI-38 cells at PDL25 through to PDL50 were able to be classified as either EP, ES, or DS (Fig. 3e; original clustering in Fig. 2a). Unsurprisingly, the PDL50 cells had the highest predicted percentage of ES cells (66.6%). The percentage of predicted PDL50 cells then incrementally decreased with decreasing PDL number, with 15.7% of PDL25 cells being predicted to be ES (Fig. 3f). This suggests that even at the earlier passages, the population of fibroblasts is heterogeneous and that individual cells are on their own trajectories. Finally, for completeness, this ML predictive model for ES, EP, and DS cells was tested on the EP and DS HDF cells alone, identifying a small percentage (10.06%) of the EP cells as early senescent (Fig. 3g). However, within the DS cells, only 0.39% were predicted to be ES, indicating that the DS cells are a more homogenous population. Intriguingly, testing the ML model on two alternative senescence triggers (oncogene-induced senescence (OIS) and paracrine senescence) [10] suggests that the time spent in senescence is perhaps more important than the trigger (Additional File 1: Fig. S3a–c). The ES classifier was able to detect increasing senescence in the OIS and paracrine groups compared to the proliferating cells (27.9% and 32.9% more, respectively), but no DS cells were detected (Additional File 1: Fig. S3c).

### Testing the models using in vivo data

**Fig. 4:**
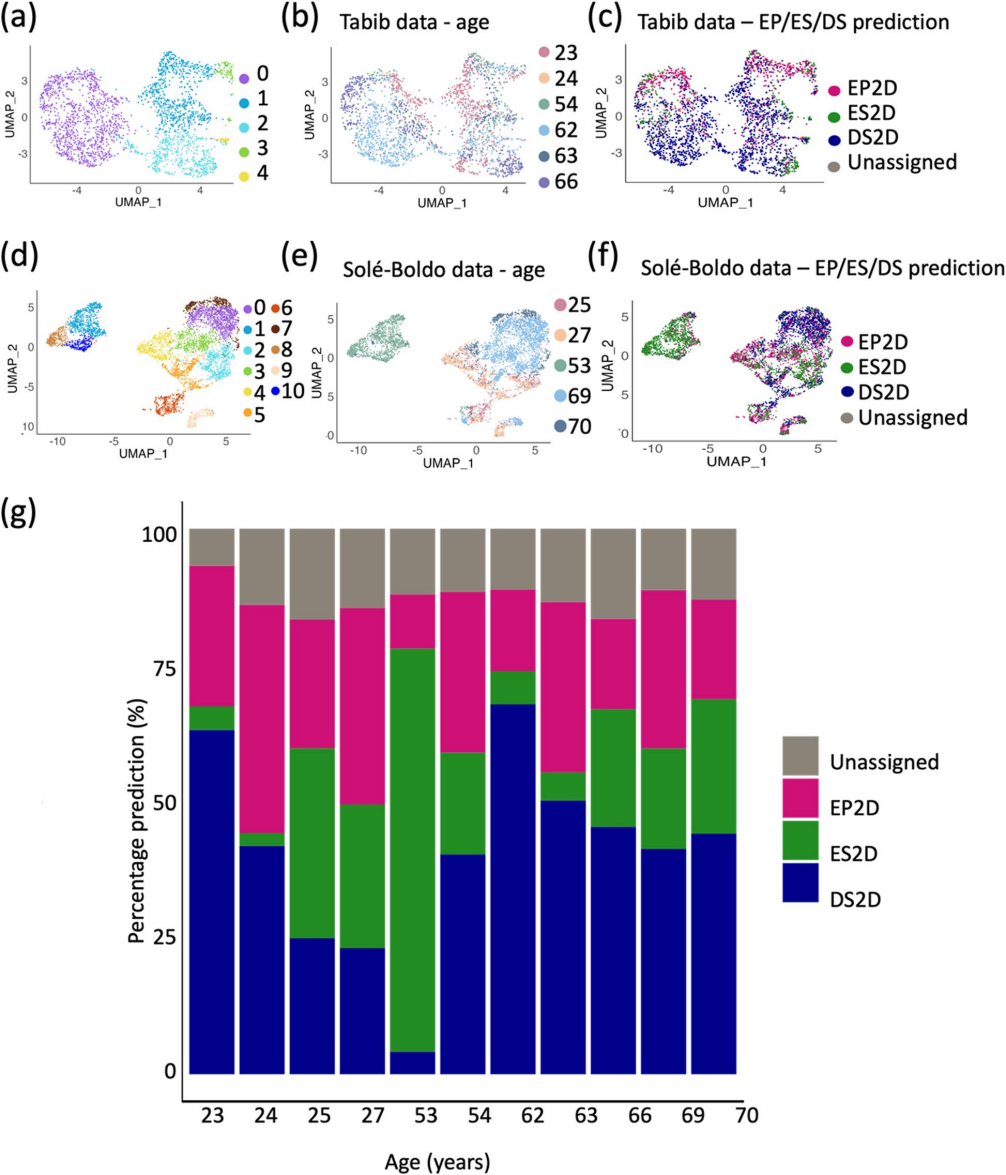
The 2D machine learning models cannot accurately be applied to in vivo datasets. **a** UMAP of scRNA-seq of in vivo fibroblasts from Tabib et al. [19]. **b** UMAP shown in **a**, overlayed with donor age. **c** UMAP shown in **a**, overlayed with MDA EP/ES/DS model prediction. **d** UMAP of scRNA-seq of in vivo fibroblasts from Solé-Boldo et al. [20]. **e** UMAP shown in **d**, overlayed with donor age. **f** UMAP shown in **d**, overlayed with MDA EP/ES/DS model prediction. **g** Barplot showing percentage model prediction of EP/ES/DS cells for each donor from both Tabib [19] and Solé-Boldo [20] datasets in **c** and **f**

To investigate whether the EP/ES/DS model can detect senescence in vivo, the models were tested on two independent publicly available whole skin scRNA-seq datasets from Tabib et al. [19] (data kindly provided by corresponding author) and Solé-Boldo et al. [20] (#GSE130973). Clustering the fibroblasts from Tabib et al. [19] revealed five clusters (Fig. 4a), which did not appear to stratify based on the age of the donors (Fig. 4b), or by their predicted senescence state (Fig. 4c). Analysis of the second whole skin scRNA-seq data from Solé-Boldo et al. [20] generated similar findings. Clustering these fibroblasts formed 11 distinct clusters (Fig. 4d), and these did appear to have some age-associated influences (Fig. 4e). Particularly, the 53-year-old donor clustered alone, which could suggest fibroblast abnormality for this individual, or perhaps issues with tissue isolation and processing. Intriguingly, this abnormality was also reflected in the percentage predictions of EP, ES, and DS cells, with the 53-year-old donor having a very high prediction of ES cells (73.9%) (Fig. 4f–g).

Combining the percentage of EP, ES and DS predictions from both the Tabib [19] and Solé-Boldo [20] datasets revealed a remarkably high prevalence of ES and DS predicted cells, regardless of donor age (Fig. 4g). The combination of DS and ES predicted cells makes up a minimum of 44.18% of the fibroblasts (24-year-old donor), and a maximum of 78.02% of the fibroblasts (53-year-old donor). Evidence within the literature suggests that senescent fibroblasts within human skin in vivo make up approximately 8–15% of the fibroblast population [36–39]. However, this literature relies on single markers which are not necessarily specific to senescence, or sufficient to detect all heterogeneous senescent populations, and making these predictions is confounded by a lack of ground truth in vivo [2]. Despite this, the large disparity between the model prediction and literature reports suggests that the model is not accurately able to identify senescent fibroblasts in vivo.

### Building in vitro 3D models improves senescent cell detection in vivo

**Fig. 5:**
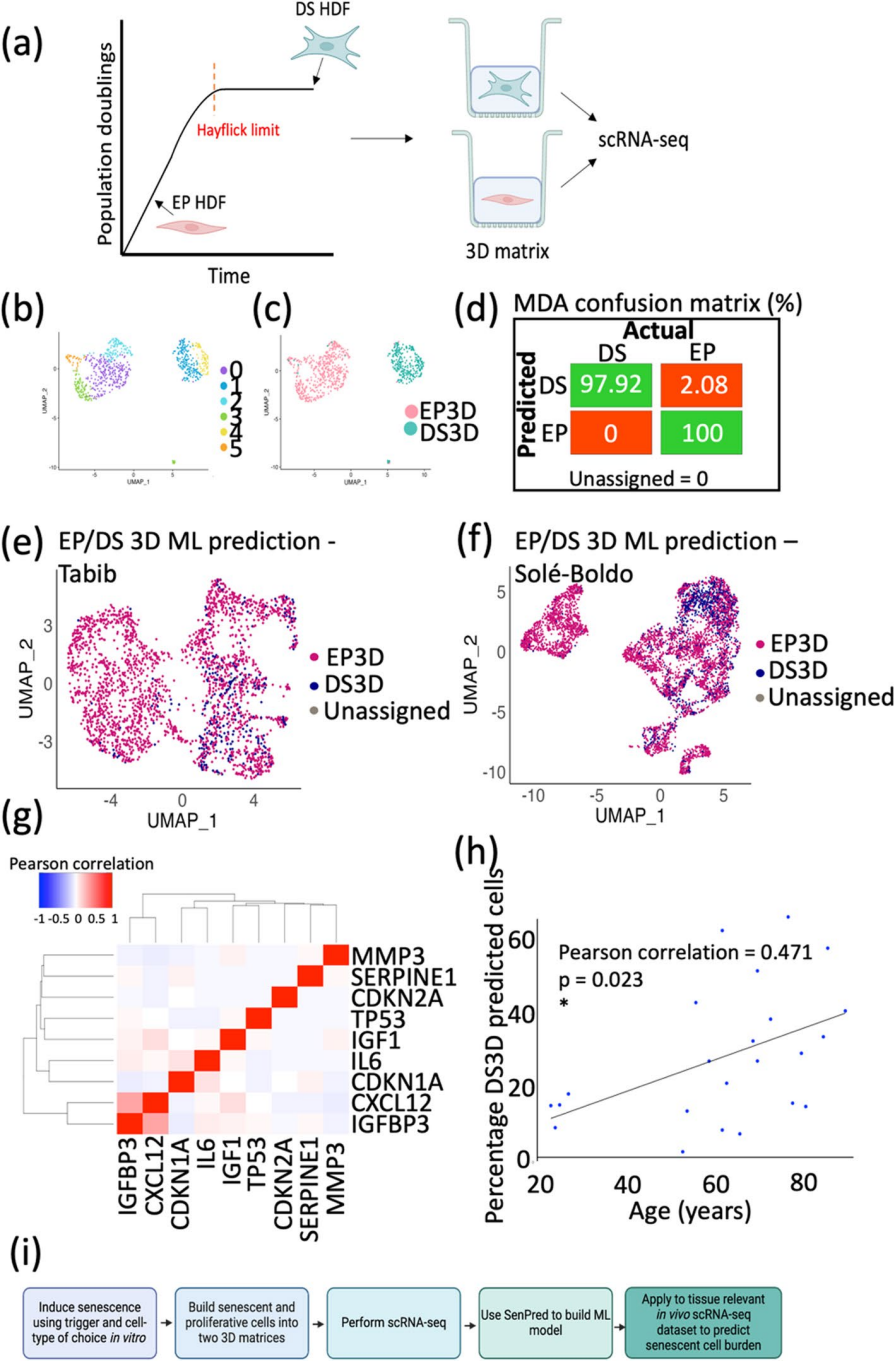
A ML model built from 3D dermal gels more accurately recapitulates senescence in vivo. **a** Schematic illustration of the position of early proliferative (EP) and deeply senescent (DS) human dermal fibroblasts (HDFs) on the Hayflick curve. The samples were grown in a 3D Matrigel-collagen matrix before single-cell RNA sequencing (scRNA-seq). Created with BioRender.com. **b** UMAP clustering of scRNA-seq of HDFs grown in a 3D matrigel matrix. **c** UMAP in **b**, overlayed with sample type. **d** Confusion matrix of MDA model of EP and DS 3D cells, highlighting true positives and true negatives (green), and false positives and false negatives (red). **e** Fig. 4a, overlayed with 3D ML model prediction. **f** UMAP in Fig. 4d, overlayed with 3D ML model prediction. **g** Pearson correlation matrix of normalised gene counts for a selected list of classic senescence markers, in the DS predicted cells from Tabib [19] and Solé-Boldo [20]. **h** Dotplot of the percentage of DS predicted cells using SenPred 3D, compared with donor age, for Tabib [19], Solé-Boldo [20], and Ganier [22] data. **i** Flowchart showing the steps of the SenPred pipeline

In recent years, the limitations of culturing cells in a two-dimensional monolayer have been widely reported. Most importantly, cells cultured in a monolayer do not replicate the complexities and depth of the extracellular matrix in vivo, which can have wide implications on cell growth and signalling processes [12–15, 40, 41]. Consequently, three-dimensional organotypic models have been developed in an effort to recapitulate in vivo cell behaviour more accurately. For this reason, we built a 3D fibroblast matrigel-collagen dermal gel containing either EP or DS HDFs, which underwent 10X scRNA-seq (Fig. 5a). To determine whether the transcriptome of fibroblasts grown in 3D or 2D was more similar to fibroblasts in vivo, a heatmap was constructed comparing the core matrisome (structural ECM proteins) between these groups (Additional File 1: Fig. S4) [42]. The core matrisome proteins measured are listed in Additional File 2; Supplementary Table S2. Hierarchical clustering reveals two initial branched clusters, where the fibroblasts grown in 2D cluster alone, and the fibroblasts grown in a 3D gel clustered alongside the in vivo fibroblasts from both Tabib et al. [19] and Solé-Boldo et al. [20], suggesting fibroblasts grown in a 3D gel are more similar to those in vivo based on their expression of ECM components.

Clustering of the fibroblasts grown in a 3D gel revealed six distinct groups, and a clear separation of EP and DS cells (Fig. 5b–c), highlighting that the transcriptomic differences between EP and DS cells were maintained in three-dimensional culture. Next, a MDA ML model was built to classify the cells from the dermal gels as EP or DS (called 3D SenPred), and intra-experimental testing revealed over 97.92% true positives (Fig. 5d). Promisingly, applying this ML model to the dataset from Tabib et al. [19] revealed a more physiological level of senescence prediction, with the maximum percentage of senescent fibroblasts being predicted in the 63-year-old (21.34%) (Fig. 5e). This was recapitulated when the 3D SenPred model was applied to the Solé-Boldo [20] dataset, with the highest predicted senescent cell burden being 32.79% in the 69-year-old donor (Fig. 5f).

Mine et al. reported that papillary fibroblasts change with age compared to reticular fibroblasts, including increasing secretion of keratinocyte growth factor, increased age-related contraction of collagen gels, and decreased population doublings and clonogenic capacity with age [43], suggesting that papillary fibroblasts are likely to be the fibroblast subtype to undergo replicative senescence in vivo. Therefore, we investigated the proportion of senescent cells within the four fibroblast subtypes defined by Solé-Boldo et al. [20]: secretory reticular, pro-inflammatory, secretory papillary, and mesenchymal. Promisingly, the secretory papillary fibroblasts from Solé-Boldo et al. [20] had the highest percentage of senescence prediction (40.25%), providing increased confidence that the ML model is detecting senescent cells in vivo (Additional File 1: Fig. S5).

Characterisation of senescence classically relies of the use of a selected panel of markers. Therefore, we were interested to explore how the expression of a panel of 9 commonly used senescence markers (CXCL12, CDKN1A (p21), CDKN2A (p16/Arf), IGF1, IGFBP3, IL-6, MMP3, SERPINE1, TP53) compared on a per cell basis. We started by using the scRNAseq data for the in vitro DS3D and EP3D cells because these have a state of “ground truth”. Using this data, we generated Pearson correlation coefficients for the comparison of normalised gene counts for each of the senescence markers, displaying the resulting correlation coefficients in a heatmap (Additional File 1: Fig. S6a–b). The highest positive correlation coefficient in the DS cells was when IGFBP3 and CXCL12 were compared (0.39), and this compared to 0.28 in the EP3D cells (Additional File 1: Fig. S6a–b).

In parallel, we generated Pearson correlation coefficients for the cells that the 3D SenPred model predicted as DS or EP within the Tabib [19] and Solé-Boldo [20] datasets (Fig. 5g and Additional File 1: Fig. S6C, respectively). Again, the highest correlation in the predicted DS cells was between IGFBP3 and CXCL12 (0.35), compared to 0.22 in the predicted EP cells. The Pearson correlation coefficient between CDKN1A and CDKN2A expression in the DS and EP predicted cells were 0.0046 and − 0.0022, respectively (individual correlation plots provided in Additional File 1: Fig. S6d–e), and between IGFBP3 and TP53 the correlation coefficient was 0.0225 and − 0.0126, respectively (individual correlation plots provided in Additional File 1: Fig. S6f–g) [22]. Further, 49.38% of gene pairs have a Pearson correlation coefficient of zero or less than zero (i.e. a negative correlation) in the DS predicted cells. Overall, this exercise revealed the absence of a strong correlation between pairs of commonly used senescence markers at the single cell level both in vivo and even in vitro where we have a state of ground truth, and emphasises how using preselected markers might only detect a subset of senescent cells. Taken together, this data further highlights the value of using SenPred, a holistic machine learning model built using thousands of genes, to detect the likelihood of a cell being senescent based on more nuanced changes, with the potential for improved identification of heterogeneous senescent cells whilst removing the likelihood of single-marker based false positives or false negatives.

Next, we expanded the in vivo datasets to incorporate a further 12 whole skin scRNA-seq dataset donors recently generated by Ganier et al. [22]. First, the fibroblasts were identified in line with Ganier et al. [22] (Additional File 1: Fig. S7). We then used the 3D SenPred model to predict the percentage of DS fibroblasts. Finally, we combined a total of 23 donor samples from the three independent in vivo datasets: Tabib [19], Solé-Boldo [20], and Ganier [22] to explore how the SenPred output changes relative to donor age (Fig. 5H). We observed that the percentage of DS predicted fibroblasts is significantly positively correlated with age (Pearson = 0.471, *p* value = 0.023), indicating that SenPred generated from 3D fibroblasts successfully predicts increasing percentages of deeply senescent fibroblast cells with age. Crucially, SenPred generated from 2D fibroblasts does not show any significant trend with age (Pearson = − 0.26, *p* = 0.23), further confirming the advantage of utilising data generated with cells grown in a 3D environment (Additional File 1: Fig. S8a). Unsurprisingly, the trend is also lost when the model is tested on whole skin (Additional File 1: Fig. S8b) or on fibroblasts isolated from lung tissue (Additional File 1: Fig. S8c) [24], highlighting the model’s tissue and cell type specificity in vivo.

In summary, the SenPred pipeline allows the generation of an unbiased predictive model of dermal fibroblast senescence. Using scRNA-seq data from cells with a known senescence classification, ML models can be generated and evaluated both intra- and inter-experimentally (Fig. 5i). In the future, these models could be generated in multiple tissues, trigger and cell-type-specific contexts of interest. The models can then be applied to classify senescence within unknown datasets. Importantly, using scRNA-seq data generated from cells grown in 3D allows the detection of senescent cells in vivo.

## Discussion

The term ‘senescence’ encompasses a wide variety of cellular outcomes, which display diverse context-dependent states [1–3]. For this reason, there is no universal marker of senescence, and we believe that characterisation and classification of senescence should focus on identifying specific senescent cell subtypes. It is also important to consider the heterogeneity of these subtypes, particularly the heterogeneity of in vivo aged tissue, where the minority of cells within the tissue are likely to be senescent [36]. The emergence of scRNA-seq datasets has allowed for a greater understanding of this heterogeneity at the transcriptomic level. However, the lack of a single established senescence marker means identifying senescent cells within these datasets is arduous and requires prior knowledge of the specific senescent cell subtype. Recently, the SenNet Consortium was established [44], with the overarching aim of providing an atlas of senescence characterisation and mapping. With the growth of this database, it is important to have a tool which will allow rapid classification of senescence based on the characterisation atlas. Although AI and ML are emerging techniques for the classification of cellular subtypes, particularly within whole tissues or disease states, to date ML has not been used to classify senescence based on scRNA-seq data. Previously published work focussed on morphological classification or using bulk RNA sequencing datasets which perhaps miss the nuanced nature of senescence at the single cell level [7–11].

Within this work, we develop a ML model which can successfully classify deeply senescent fibroblasts in both internal and external in vitro datasets. Intriguingly, the depth of senescence appears more important than the trigger type when applying the model in vitro, as an early senescent classifier can detect oncogene-induced and paracrine senescent cells. Applying the ML model based on scRNA-seq from fibroblasts grown in 2D was unable to successfully determine senescence in fibroblasts from in vivo tissues, predicting an unrealistic senescent cell burden. Sequencing cells which were grown in a 3D environment more closely recapitulated reported senescent cell numbers in vivo and also detected senescence in the expected fibroblast subtypes. This suggests that future efforts to further characterise senescent cell heterogeneity using scRNAseq should consider harvesting cells following 3D rather than 2D culture. However, it is equally important to note that there are numerous environmental differences between cells grown in 3D and those in in vivo [12–15].

Current recommendations are to profile senescent cells using a panel of selected senescence markers. When we asked how these markers correlated in vitro (where the ground truth was known) and in vivo at the single cell level we found that the degree of positive correlation was at best 0.39 in vitro and 0.35 in vivo, with the majority of the marker pairs showing weak or negative correlation. This observation underscores the transcriptional heterogeneity of senescence at the single-cell level and the potential for bias in senescent cell identification based on a small marker panel. It also highlights the advantage of using the SenPred pipeline, which allows the detection of numerous, holistic, and bidirectional changes across 2,000 genes, enabling the detection of heterogeneous senescent cell subgroups which could otherwise be overlooked.

Using currently available scRNAseq datasets, we were able to detect a significantly increasing percentage of predicted fibroblast senescence with age. Importantly, when comparing SenPred with other correlation-based skin ageing markers, SenPred was able to reach a similar and significant positive correlation with a smaller sample size (Pearson = 0.471, *p* value − 0.023, 23 donors). For example, the work by Demanelis et al. used 286 independent donors to explore the relationship between telomere length and age in sun-unexposed skin, generating a significant negative Pearson correlation of -0.26 [45]. This comparison highlights a potential strength of the SenPred approach, whereby the use of a large number of markers to build a model could increase accuracy, reduce the influence of biological variation and potentially reduce the number of donors required to conduct future studies. Age-related correlation data inevitably contains biological variability. In the future, the ability to carry out physiological assessments of the donors could also strengthen the approach and allow for the comparison of biological versus chronological age. The existence of survivor bias should also be considered, whereby a high senescent cell burden is likely associated with increased mortality.

The evaluation of model performance in vivo represents a clear limitation of this study. At present, the full spectrum of in vivo triggers of senescence and thus potential signatures have yet to be elucidated, and thus the state of “ground truth” is currently unknown. Consequently, there is somewhat of a catch-22 when attempting to truly define senescent cell burden in vivo. The opportunity to use spatial transcriptomics alongside a classic senescence marker, although perhaps reductive based on the nuanced nature of senescence, may allow a more accurate evaluation of model performance in vivo. The advantages of multi-modal AI could be further explored, combining multiple ‘omics’ datasets to improve senescence prediction.

## Conclusions

In summary, we position SenPred as a proof-of-concept pipeline, with the future aspiration to build a more holistic ML model, whereby the model could detect multiple subtypes of senescence based on multiple triggers and cell types. Presently, it is unclear whether the 3D SenPred model can detect multiple triggers in vivo, but the model does appear to be cell-type and tissue specific. Currently, the datasets to build the model with multiple triggers and cell types are unavailable, but we envision this to change in line with the goals of the SenNet consortium [44]. The emergence of this novel data will allow continuous model testing and improvement, increasing confidence in the predictive power of SenPred. It should be noted, however, that this work has been generated using adult replicative senescent cells, arguably the most experimentally challenging model, and therefore implementation with other triggers should be more straightforward for others to build upon.

A holistic senescence prediction model would have multiple clinical benefits, including predicting individual patient senescent cell burden and trajectory, and the likelihood of senescence-associated age-related diseases. This could enable prediction of patient lifespan, personalised, early interventions, and potential efficacy of therapeutic interventions. For example, the model could be used to test senolytic activity by measuring the clearance of senescent cells. This would support work by Smer-Barreto et al., using ML to discover novel senolytics [46]. The SenPred workflow has potential wider utility, as it could be applied to additional cellular contexts and disease states beyond senescence, simply requiring scRNA sequencing of cells with context-specific ground truth. This would allow the detection of heterogeneous cells within tissues, prediction of disease burden, and application and evaluation of personalised medicines. In conclusion, we believe ML is a valuable tool which can contribute towards the collective goal of the senescence field to characterise and classify senescence and could have wider application across numerous disease states.

## Supplementary Information

Additional file 1: Supplementary Figures S1-S8 with figure legends and Supplementary Table S1.

Additional file 2: Supplementary Table S2, a List of matrisome genes used to cluster samples in Figure S4.

## Data Availability

Package dependencies All packages and versions used in this code are available at the following URL: https://github.com/bethk-h/SenPred_HDF [47]. Data Availability The scRNAseq data generated as part of this work has been uploaded to GEO #GSE282425 [48]. Code Availability All code for this project is accessible via the following URL: https://github.com/bethk-h/SenPred_HDF [47].

## References

[1] Hernandez-Segura A, Nehme J, Demaria M. Hallmarks of cellular senescence. Trends Cell Biol. 2018;28:436–53. 29477613 10.1016/j.tcb.2018.02.001

[2] González-Gualda E, Baker AG, Fruk L, Muñoz-Espín D. A guide to assessing cellular senescence in vitro and in vivo. FEBS J. 2021;288:56–80. 32961620 10.1111/febs.15570

[3] Huang W, Hickson LJ, Eirin A, Kirkland JL, Lerman LO. Cellular senescence: the good, the bad and the unknown. Nat Rev Nephrol. 2022;18:611–27. 35922662 10.1038/s41581-022-00601-z PMC9362342

[4] Kohli J, et al. Algorithmic assessment of cellular senescence in experimental and clinical specimens. Nat Protoc. 2021;16:2471–98. 33911261 10.1038/s41596-021-00505-5 PMC8710232

[5] Hughes BK, Wallis R, Bishop CL. Yearning for machine learning: applications for the classification and characterisation of senescence. Cell Tissue Res. 2023;394:1–16. 37016180 10.1007/s00441-023-03768-4 PMC10558380

[6] Sidey-Gibbons JAM, Sidey-Gibbons CJ. Machine learning in medicine: a practical introduction. BMC Med Res Methodol. 2019;19:1–18. 30890124 10.1186/s12874-019-0681-4 PMC6425557

[7] Heckenbach I. et al. Nuclear morphology is a deep learning biomarker of cellular senescence; 2022. 10.1038/s43587-022-00263-3 PMC10154217 37118134

[8] Saul D, et al. A new gene set identifies senescent cells and predicts senescence-associated pathways across tissues. Nat Commun. 2022;13:4827. 35974106 10.1038/s41467-022-32552-1 PMC9381717

[9] Khadirnaikar S, Chatterjee A, Shukla S. Identification and characterization of senescence phenotype in lung adenocarcinoma with high drug sensitivity. Am J Pathol. 2021;191:1966–73. 34358516 10.1016/j.ajpath.2021.07.005

[10] Teo YV, et al. Notch signaling mediates secondary senescence. Cell Rep. 2019;27:997–1007. 31018144 10.1016/j.celrep.2019.03.104 PMC6486482

[11] Jochems F, et al. The cancer SENESCopedia: a delineation of cancer cell senescence. Cell Rep. 2021;36:109441. 34320349 10.1016/j.celrep.2021.109441 PMC8333195

[12] Kular JK, Basu S, Sharma RI. The extracellular matrix: Structure, composition, age-related differences, tools for analysis and applications for tissue engineering. J Tissue Eng. 2014;5:2041731414557112. 25610589 10.1177/2041731414557112 PMC4883592

[13] Reijnders CMA, et al. Development of a full-thickness human skin equivalent in vitro model derived from TERT-immortalized keratinocytes and fibroblasts. Tissue Eng Part A. 2015;21:2448–59. 26135533 10.1089/ten.tea.2015.0139 PMC4554934

[14] Weinmüllner R, et al. Organotypic human skin culture models constructed with senescent fibroblasts show hallmarks of skin aging. NPJ Aging Mech Dis. 2020;6:4. 32194977 10.1038/s41514-020-0042-x PMC7060247

[15] Stabell AR, et al. Single-cell transcriptomics of human-skin-equivalent organoids. Cell Rep. 2023;42:112511. 37195865 10.1016/j.celrep.2023.112511 PMC10348600

[16] Chan M, et al. Novel insights from a multiomics dissection of the Hayflick limit. Elife. 2022;11:e70283. 35119359 10.7554/eLife.70283 PMC8933007

[17] Chan M, Yuan H, Soifer I & M Maile T. Novel insights from a multiomics dissection of the Hayflick limit. Gene Expression Omnibus; 2022. https://www.ncbi.nlm.nih.gov/geo/query/acc.cgi?acc=GSE175533 10.7554/eLife.70283 PMC8933007 35119359

[18] Teo YV. et al. Notch signaling mediates secondary senescence. Gene Expression Omnibus; 2019. 10.1016/j.celrep.2019.03.104. https://www.ncbi.nlm.nih.gov/geo/query/acc.cgi?acc=GSE115301 10.1016/j.celrep.2019.03.104 PMC6486482 31018144

[19] Tabib T, Morse C, Wang T, Chen W, Lafyatis R. SFRP2/DPP4 and FMO1/LSP1 define major fibroblast populations in human skin. J Investig Dermatol. 2018;138:802–10. 29080679 10.1016/j.jid.2017.09.045 PMC7444611

[20] Solé-Boldo L, et al. Single-cell transcriptomes of the human skin reveal age-related loss of fibroblast priming. Commun Biol. 2020;3:1–12. 32327715 10.1038/s42003-020-0922-4 PMC7181753

[21] Solé-Boldo L. et al. Single-cell transcriptomes of the human skin reveal age-related loss of fibroblast priming. Gene Expression Omnibus; 2020 10.1038/s42003-020-0922-4https://www.ncbi.nlm.nih.gov/geo/query/acc.cgi?acc=GSE130973 10.1038/s42003-020-0922-4 PMC7181753 32327715

[22] Ganier C, et al. Multiscale spatial mapping of cell populations across anatomical sites in healthy human skin and basal cell carcinoma. Proc Natl Acad Sci. 2024;121:e2313326120. 38165934 10.1073/pnas.2313326120 PMC10786309

[23] Ganier C. et al. Multiscale spatial mapping of cell populations across anatomical sites in healthy human skin and basal cell carcinoma. ArrayExpress2024. 10.1073/pnas.2313326120https://www.ebi.ac.uk/biostudies/arrayexpress/studies/E-MTAB-13085 10.1073/pnas.2313326120 PMC10786309 38165934

[24] Sikkema L, et al. An integrated cell atlas of the lung in health and disease. Nat Med. 2023;29:1563–77. 37291214 10.1038/s41591-023-02327-2 PMC10287567

[25] Sikkema L. et al. An integrated cell atlas of the lung in health and disease. CZ CellxGene Discover; 2023 10.1038/s41591-023-02327-2. https://cellxgene.cziscience.com/collections/6f6d381a-7701-4781-935c-db10d30de293 10.1038/s41591-023-02327-2 PMC10287567 37291214

[26] Stuart T, et al. Comprehensive integration of single-cell data. Cell. 2019;177:1888–902. 31178118 10.1016/j.cell.2019.05.031 PMC6687398

[27] Hafemeister C, Satija R. Normalization and variance stabilization of single-cell RNA-seq data using regularized negative binomial regression. Genome Biol. 2019;20:296. 31870423 10.1186/s13059-019-1874-1 PMC6927181

[28] Hao Y, et al. Integrated analysis of multimodal single-cell data. Cell. 2021;184:3573–87. 34062119 10.1016/j.cell.2021.04.048 PMC8238499

[29] Zappia L, Oshlack A. Clustering trees: a visualization for evaluating clusterings at multiple resolutions. Gigascience. 2018;7:giy083. 30010766 10.1093/gigascience/giy083 PMC6057528

[30] Alquicira-Hernandez J, Sathe A, Ji HP, Nguyen Q, Powell JE. scPred: accurate supervised method for cell-type classification from single-cell RNA-seq data. Genome Biol. 2019;20:1–17. 31829268 10.1186/s13059-019-1862-5 PMC6907144

[31] Cao J, et al. The single-cell transcriptional landscape of mammalian organogenesis. Nature. 2019;566:496–502. 30787437 10.1038/s41586-019-0969-x PMC6434952

[32] Qiu X, et al. Reversed graph embedding resolves complex single-cell trajectories. Nat Methods. 2017;14:979–82. 28825705 10.1038/nmeth.4402 PMC5764547

[33] Trapnell C, et al. The dynamics and regulators of cell fate decisions are revealed by pseudotemporal ordering of single cells. Nat Biotechnol. 2014;32:381–6. 24658644 10.1038/nbt.2859 PMC4122333

[34] Wallis R, et al. Senescence-associated morphological profiles (SAMPs): an image-based phenotypic profiling method for evaluating the inter and intra model heterogeneity of senescence. Aging (Albany NY). 2022;14:4220. 35580013 10.18632/aging.204072 PMC9186762

[35] Alcorta DA, et al. Involvement of the cyclin-dependent kinase inhibitor p16 (INK4a) in replicative senescence of normal human fibroblasts. Proc Natl Acad Sci. 1996;93:13742–7. 8943005 10.1073/pnas.93.24.13742 PMC19411

[36] Xu M, et al. Senolytics improve physical function and increase lifespan in old age. Nat Med. 2018;24:1246–56. 29988130 10.1038/s41591-018-0092-9 PMC6082705

[37] Dimri GP, et al. A biomarker that identifies senescent human cells in culture and in aging skin in vivo. Proc Natl Acad Sci. 1995;92:9363–7. 7568133 10.1073/pnas.92.20.9363 PMC40985

[38] Ressler S, et al. p16INK4A is a robust in vivo biomarker of cellular aging in human skin. Aging Cell. 2006;5:379–89. 16911562 10.1111/j.1474-9726.2006.00231.x

[39] Idda ML, et al. Survey of senescent cell markers with age in human tissues. Aging (Albany NY). 2020;12:4052. 32160592 10.18632/aging.102903 PMC7093180

[40] Duval K, et al. Modeling physiological events in 2D vs. 3D cell culture. Physiology. 2017;32:266–77. 28615311 10.1152/physiol.00036.2016 PMC5545611

[41] Baker BM, Chen CS. Deconstructing the third dimension–how 3D culture microenvironments alter cellular cues. J Cell Sci. 2012;125:3015–24. 22797912 10.1242/jcs.079509 PMC3434846

[42] Petrov PB, Considine JM, Izzi V, Naba A. Matrisome AnalyzeR–a suite of tools to annotate and quantify ECM molecules in big datasets across organisms. J Cell Sci. 2023;136:jcs261255. 37555624 10.1242/jcs.261255 PMC10499032

[43] Mine S, Fortunel NO, Pageon H, Asselineau D. Aging alters functionally human dermal papillary fibroblasts but not reticular fibroblasts: a new view of skin morphogenesis and aging. PLoS ONE. 2008;3:e4066. 19115004 10.1371/journal.pone.0004066 PMC2605251

[44] SenNet Consortium. NIH SenNet Consortium to map senescent cells throughout the human lifespan to understand physiological health. Nat Aging. 2022;2:1090–100. 36936385 10.1038/s43587-022-00326-5 PMC10019484

[45] Demanelis K, et al. Determinants of telomere length across human tissues. Science (1979). 2020;369:eaaz6876. 10.1126/science.aaz6876 PMC8108546 32913074

[46] Smer-Barreto V, et al. Discovery of senolytics using machine learning. Nat Commun. 2023;14:3445. 37301862 10.1038/s41467-023-39120-1 PMC10257182

[47] Hughes BK. et al. SenPred: A single-cell RNA sequencing-based machine learning pipeline to classify deeply senescent dermal fibroblast cells for the detection of an in vivo senescent cell burden . Github; 2024. https://github.com/bethk-h/SenPred_HDF 10.1186/s13073-024-01418-0 PMC11731430 39810225

[48] Hughes BK. et al. SenPred: A single-cell RNA sequencing-based machine learning pipeline to classify deeply senescent dermal fibroblast cells for the detection of an in vivo senescent cell burden . Gene Expression Omnibus; 2024. https://www.ncbi.nlm.nih.gov/geo/query/acc.cgi?acc=GSE282425 10.1186/s13073-024-01418-0 PMC11731430 39810225

