# Supplementary material for: SenPred: a single-cell RNA sequencing-based machine learning pipeline to classify deeply senescent dermal fibroblast cells for the detection of an in vivo senescent cell burden
Source: Genome Med. 2025 Jan 14;17:2. doi: 10.1186/s13073-024-01418-0 (PMC11731430; doi:10.1186/s13073-024-01418-0)
Supplement: Supplementary file 1 — Additional file 1: Supplementary Figures S1-S8 with figure legends and Supplementary Table S1. [file 13073_2024_1418_MOESM1_ESM.pdf]

Supplementary Figures

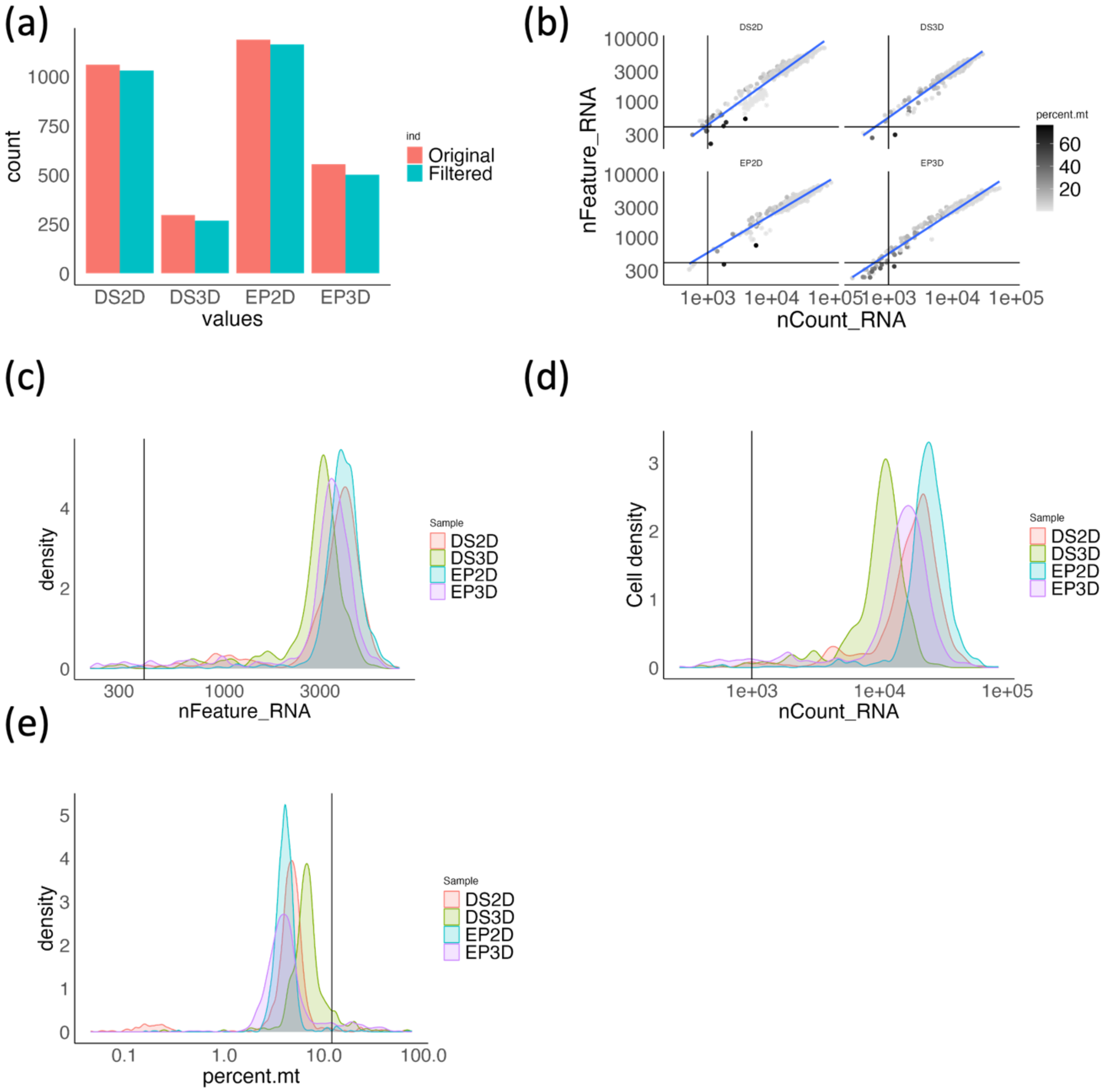

Fig S1. Quality control and filtering of scRNA-seq. (a) Number of cells sequenced before and after filtering. (b) Number of genes (nFeature_RNA) versus number of unique molecular identifiers (UMIs) (nCount_RNA), coloured by the percentage of mitochondrial RNA. (c) Density plot of nFeature_RNA for all samples. (d) Density plot of nCount_RNA for all samples. (e) Density plot of the percentage.mt for all samples. The black line represents chosen filtering cut offs in all cases.

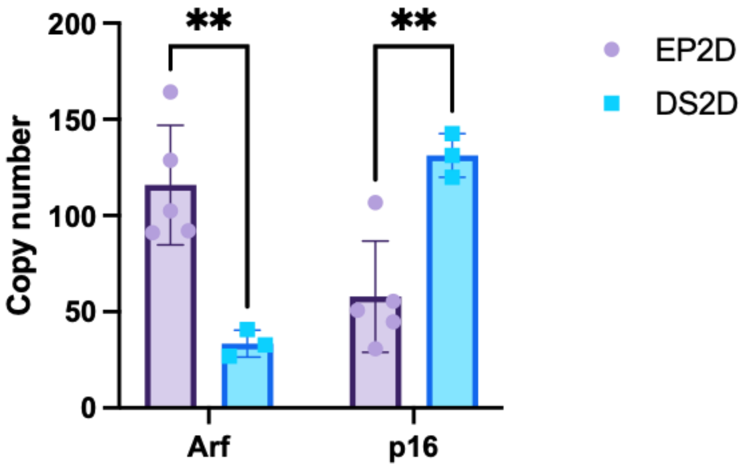

8 **Fig S2. Digital PCR measuring ARF and p16 mRNA in Early Proliferative (EP) and Deeply Senescent (DS) Human Dermal Fibroblasts (HDFs).** Copy number is normalised to 1ng/mL of cDNA loaded. Stats represents two-way ANOVA with Tukey's multiple comparison. N=5 EP, N=3 DS.

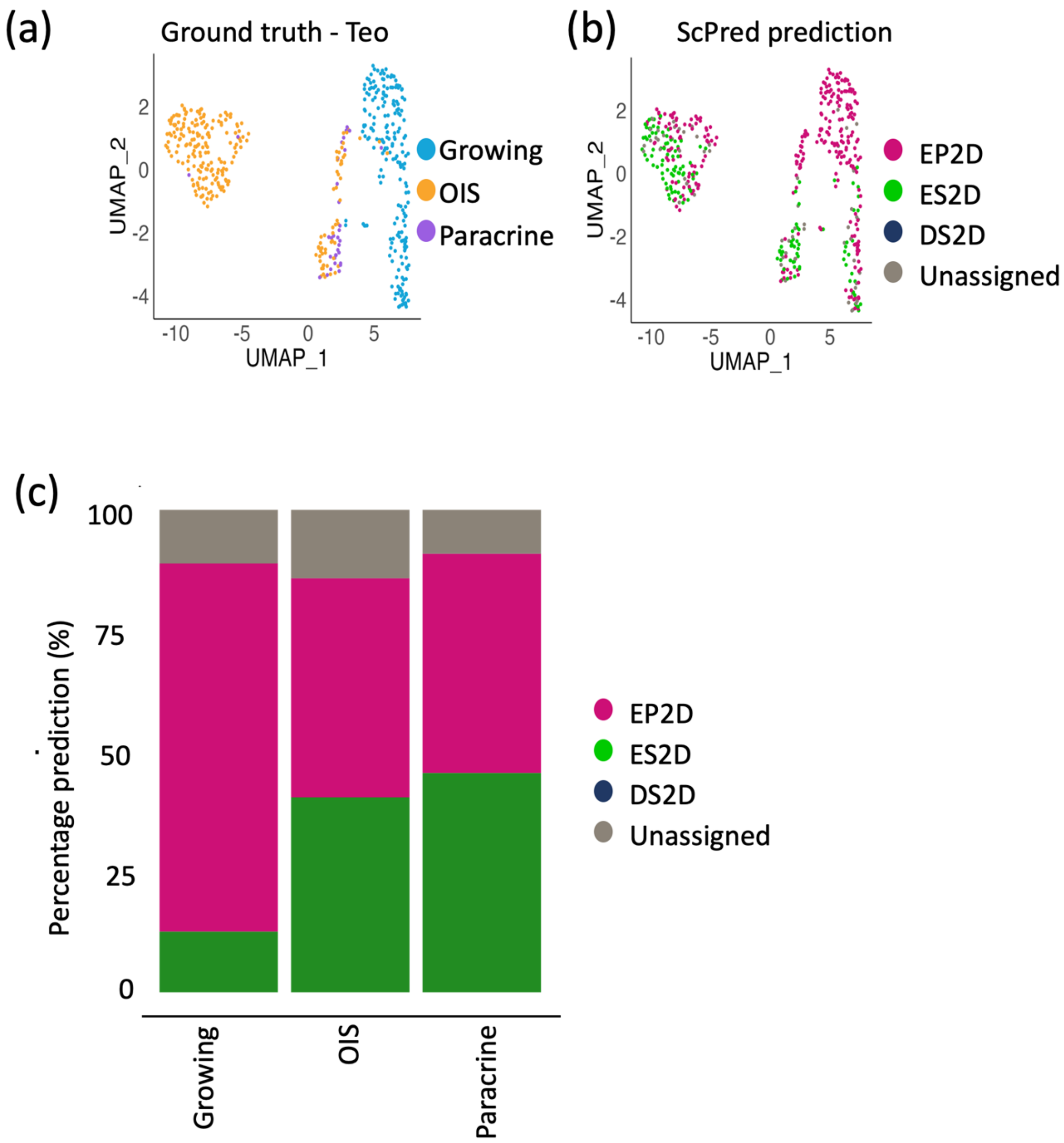

Fig S3. Applying 2D SenPred to paracrine and Oncogene Induced Senescent (OIS) cells detects early senescence. (a) UMAP of IMR90 fibroblasts from Teo et al.[19], overlayed with senescence condition. (b) UMAP in (a), overlayed with SenPred prediction. (c) Percentage of predicted Early Proliferative 2D (EP2D), Early Senescent 2D (ES2D), Deeply Senescent 2D (DS2D) and Unassigned cells in each senescence condition.

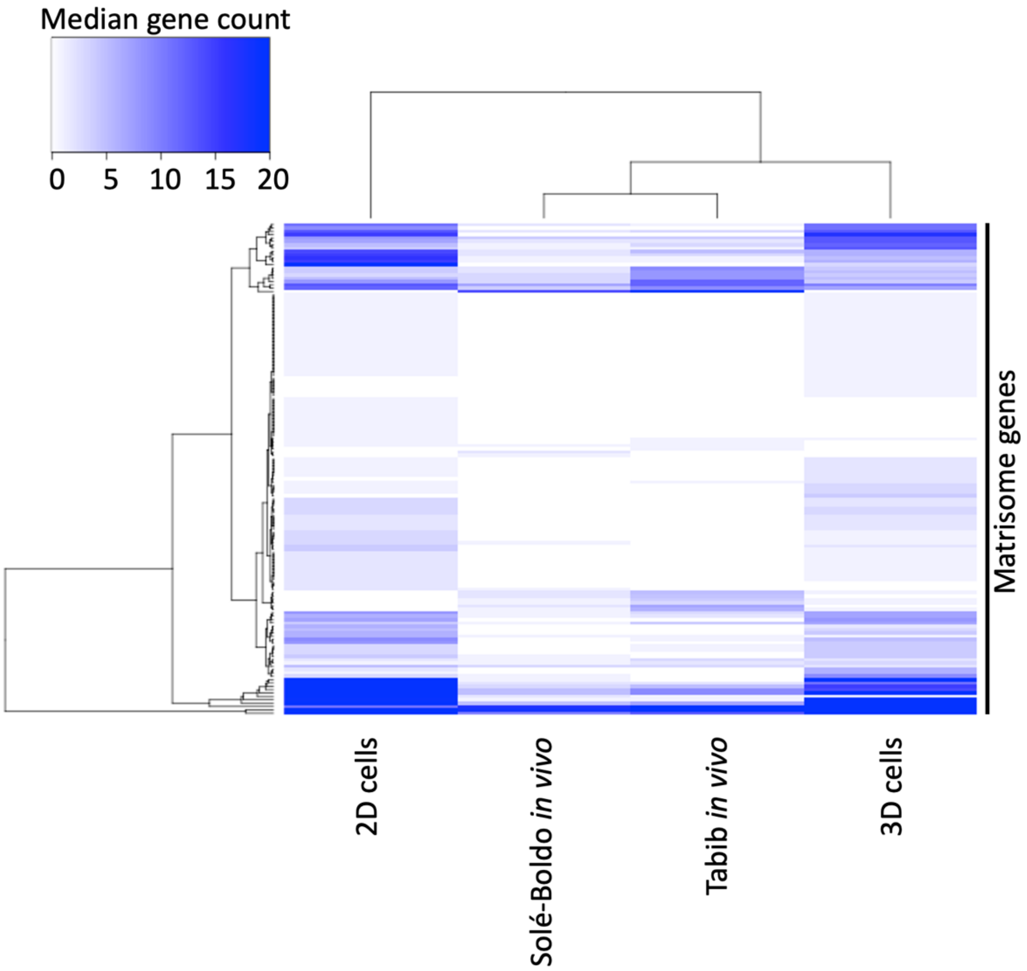

Fig S4. Clustering of matrisome gene expression reveals that fibroblasts grown in 3D are more similar to *in vivo* fibroblasts from Tabib[20] and Solé-Boldo[21] datasets, than fibroblasts grown in 2D. Heatmap displays median log-normalised gene counts for each condition, for matrisome genes listed in Supplementary List 1.

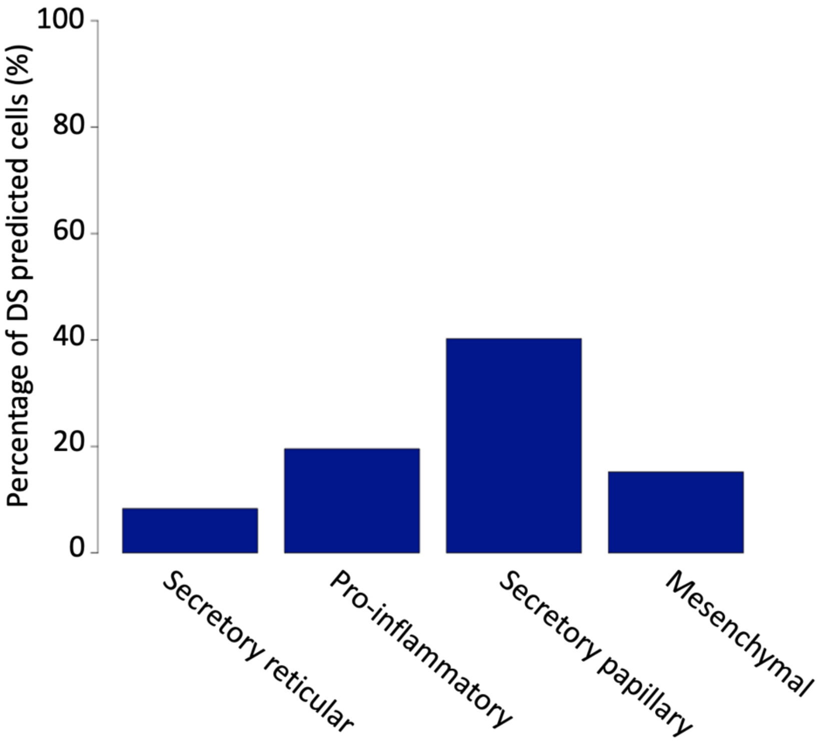

Fig S5. Barplot showing percentage of predicted deeply senescent cells in dataset from Solé-Boldo *et al.*[21] for each fibroblast subtype.

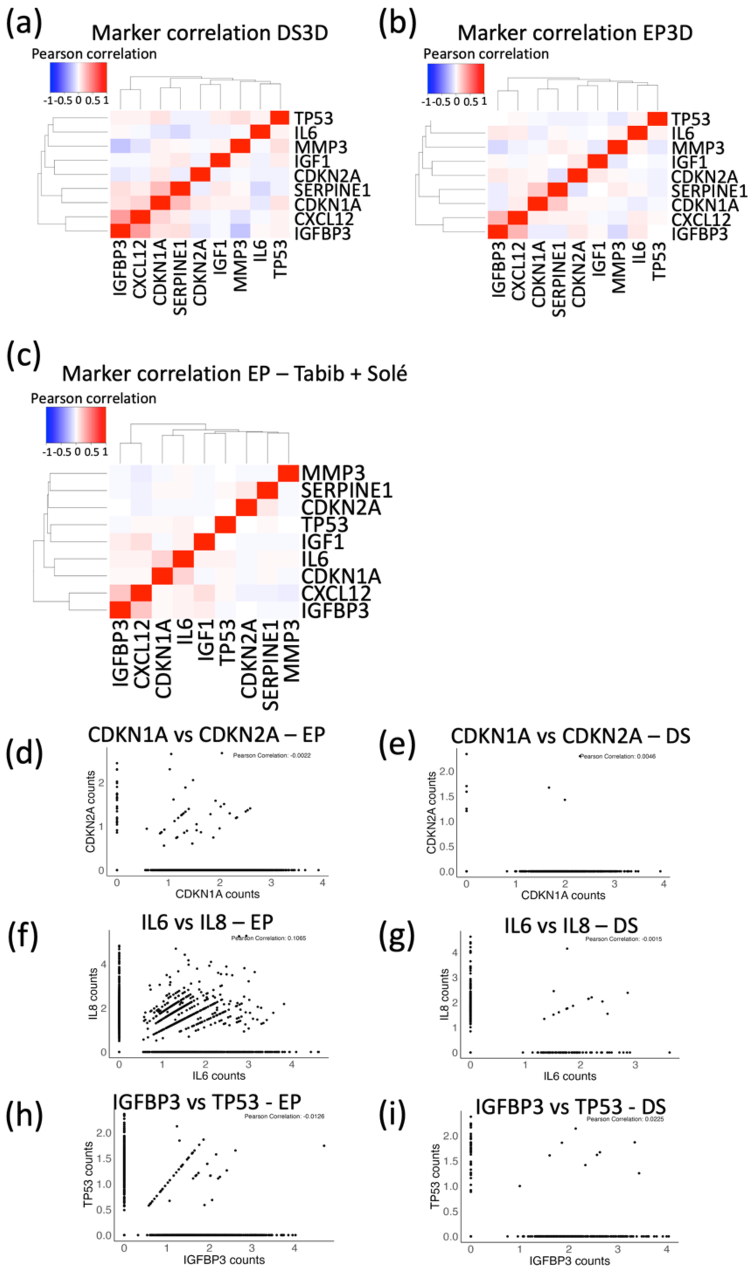

Fig S6. Pearson correlation of selected senescence markers in both Early Proliferative (EP) and Deeply Senescent (DS) cells in the 3D data, and Early Proliferative predicted cells in the Tabib[20] and Solé-Boldo[21] datasets. (a) Pearson correlation matrix of normalised gene counts for a selected list of classic senescence markers, in the DS3D cells which have a state of ground truth. (b) Pearson correlation matrix of normalised gene counts for a selected list of classic senescence markers, in the EP3D cells which have a state of ground truth. (c) Pearson correlation matrix of normalised gene counts for a selected list of classic senescence markers, in the EP predicted cells from Tabib20 and Solé-Boldo21. (b) Scatterplot of CDKN1A and CDKN2A counts of EP cells from Tabib20 and Solé-Boldo21 datasets. (c) of Scatterplot CDKN1A and CDKN2A counts of DS cells from Tabib20 and Solé-Boldo21 datasets. (d) Scatterplot of IL6 and IL8 counts of EP cells from Tabib20 and Solé-Boldo21 datasets. (e) Scatterplot of IL6 and IL8 counts of DS cells from Tabib20 and Solé-Boldo21 datasets. (f)

Scatterplot of IGFBP3 and TP53 counts of EP cells from Tabib20 and Solé-Boldo21 datasets. (g) Scatterplot of IGFBP3 and TP53 counts of DS cells from Tabib20 and Solé-Boldo21 datasets.

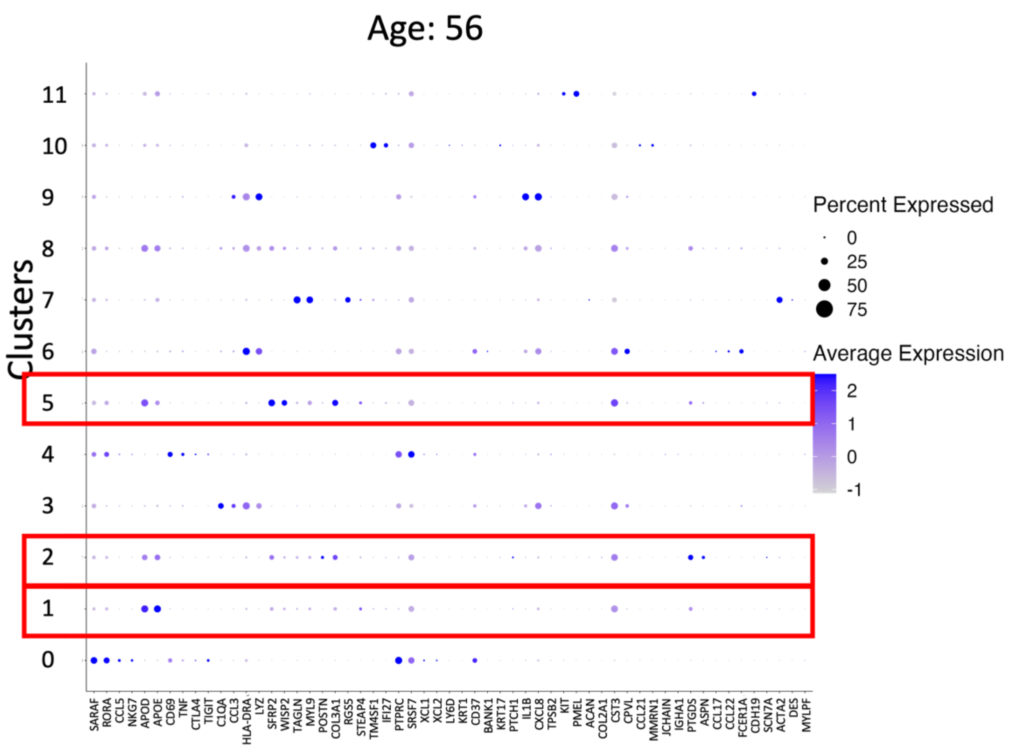
Age: 56
Clusters
11
10
9
8
7
6
5
4
3
2
1
0
SARAF RORA CCL5 NKG7 APOD APOE CD69 TNF CTLA4 TIGIT C1QA CCL3 HLA-DRA LYZ SFRP2 WISP2 TAGLN MYL9 POSTN COL3A1 RGS5 STEAP4 TM4SF1 IFI27 PTPRC SRSF7 XCL1 XCL2 LY6D KRT1 CD37 BANK1 KRT17 PTCH1 IL1B CXCL8 TPSB2 KIT PMEL ACAN COL2A1 CST3 CPVL CCL21 MMRN1 JCHAIN IGHA1 PTGDS ASPN CCL17 CCL22 FCER1A CDH19 SCN7A ACTA2 DES MYLPF
Percent Expressed
0
25
50
75
Average Expression
2
1
0
-1

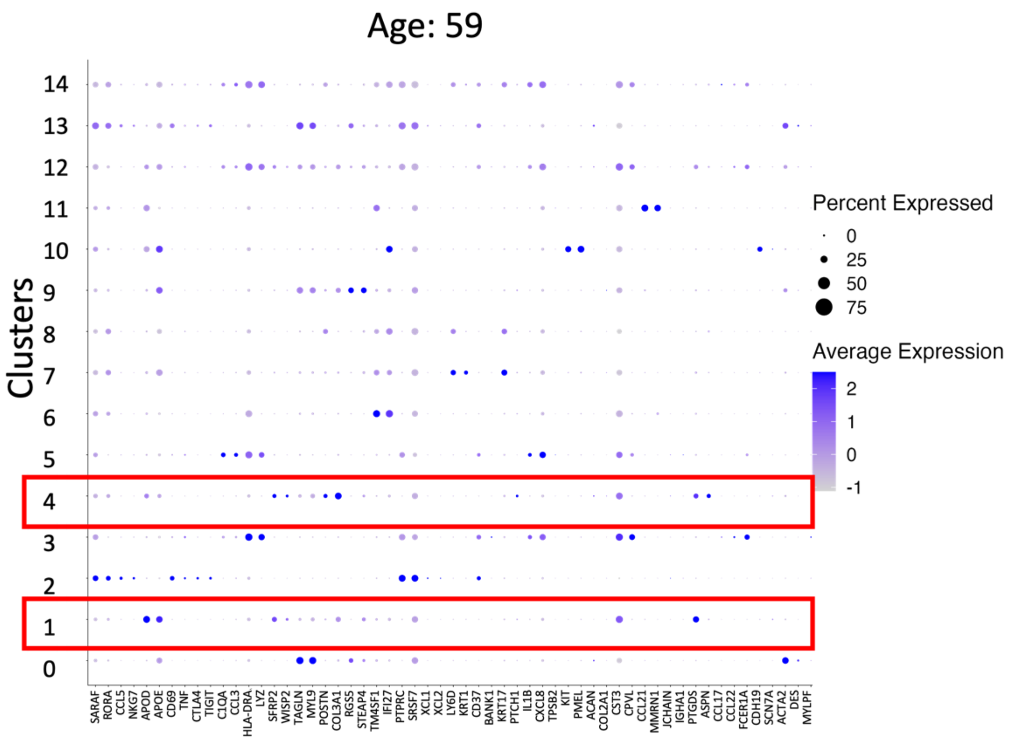
Age: 59
Clusters
14
13
12
11
10
9
8
7
6
5
4
3
2
1
0
SARAF RORA CCL5 NKG7 APOD APOE CD69 TNF CTLA4 TIGIT C1QA CCL3 HLA-DRA LYZ SFRP2 WISP2 TAGLN MYL9 POSTN COL3A1 RGS5 STEAP4 TM4SF1 IFI27 PTPRC SRSF7 XCL1 XCL2 LY6D KRT1 CD37 BANK1 KRT17 PTCH1 IL1B CXCL8 TPSB2 KIT PMEL ACAN COL2A1 CST3 CPVL CCL21 MMRN1 JCHAIN IGHA1 PTGDS ASPN CCL17 CCL22 FCER1A CDH19 SCN7A ACTA2 DES MYLPF
Percent Expressed
0
25
50
75
Average Expression
2
1
0
-1

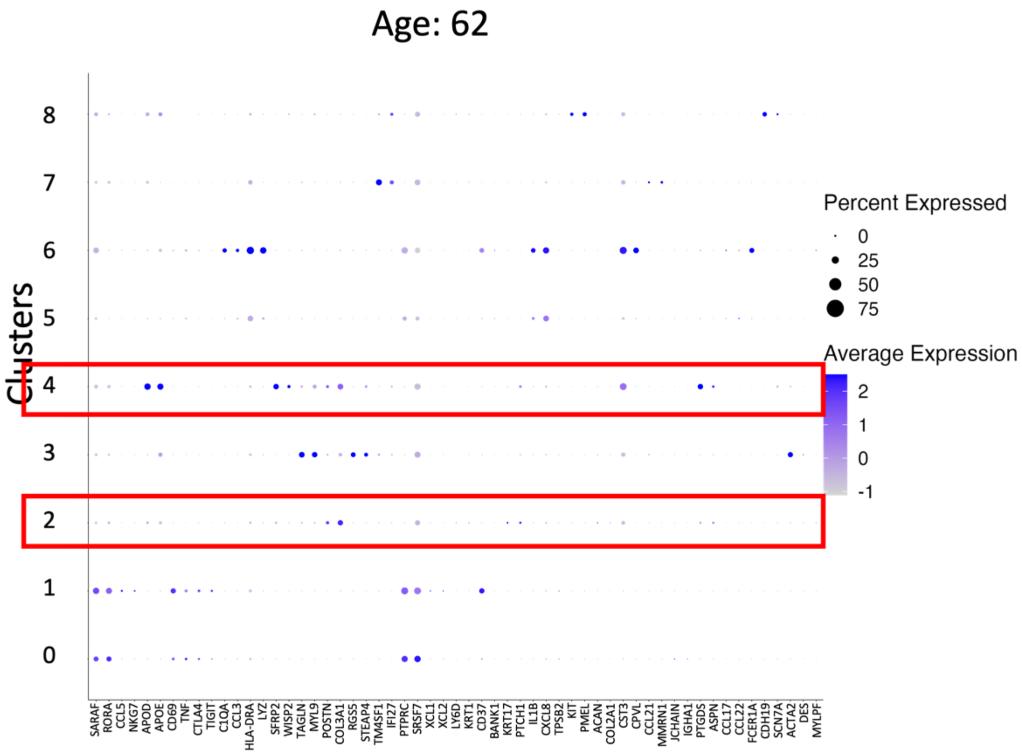
Age: 62
Clusters
8
7
6
5
4
3
2
1
0
SARAF RORA CCL5 NKG7 APOD APOE CD69 TNF CTLA4 TIGIT C1QA CCL3 HLA-DRA LYZ SFRP2 WISP2 TAGLN MYL9 POSTN COL3A1 RGS5 STEAP4 TM4SF1 IFI27 PTPRC SRSF7 XCL1 XCL2 LY6D KRT1 CD37 BANK1 KRT17 PTCH1 IL1B CXCL8 TPSB2 KIT PMEL ACAN COL2A1 CST3 CPVL CCL21 MMRN1 JCHAIN IGHA1 PTGDS ASPN CCL17 CCL22 FCER1A CDH19 SCN7A ACTA2 DES MYLPF
Percent Expressed
0
25
50
75
Average Expression
2
1
0
-1

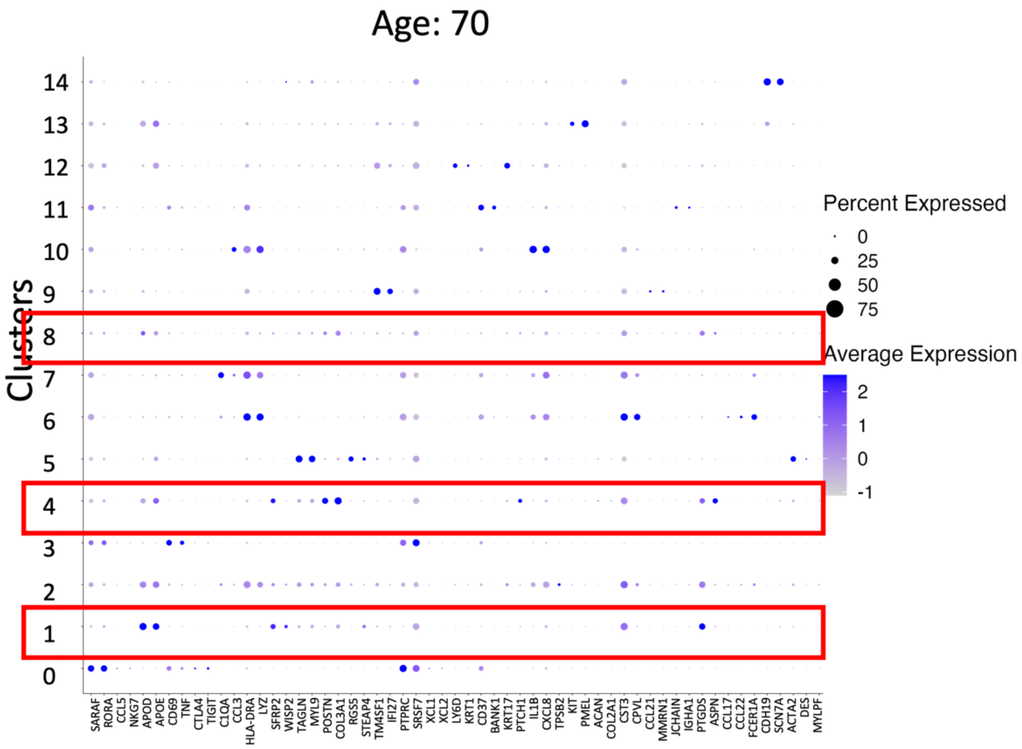
Age: 70
Clusters
14
13
12
11
10
9
8
7
6
5
4
3
2
1
0
SARAF RORA CCL5 NKG7 APOD APOE CD69 TNF CTLA4 TIGIT C1QA CCL3 HLA-DRA LYZ SFRP2 WISP2 TAGLN MYL9 POSTN COL3A1 RGS5 STEAP4 TM4SF1 IFI27 PTPRC SRSF7 XCL1 XCL2 LY6D KRT1 CD37 BANK1 KRT17 PTCH1 IL1B CXCL8 TPSB2 KIT PMEL ACAN COL2A1 CST3 CPVL CCL21 MMRN1 JCHAIN IGHA1 PTGDS ASPN CCL17 CCL22 FCER1A CDH19 SCN7A ACTA2 DES MYLPF
Percent Expressed
0
25
50
75
Average Expression
2
1
0
-1

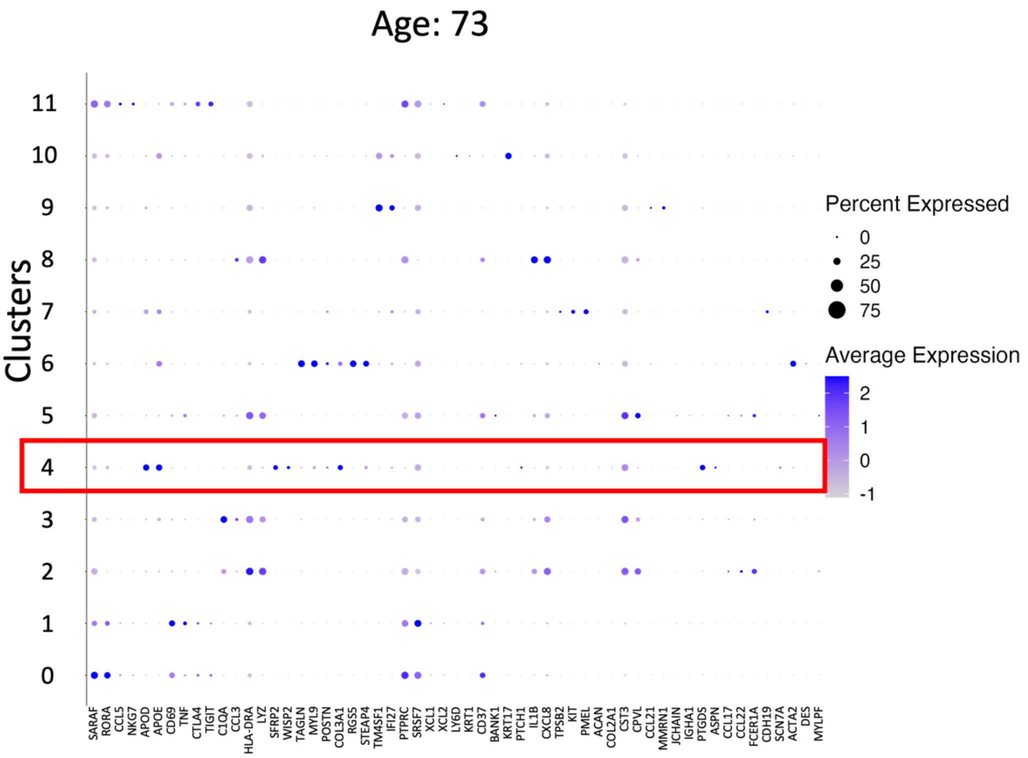
Age: 73
Clusters
11
10
9
8
7
6
5
4
3
2
1
0
SARAF RORA CCL5 NKG7 APOD APOE CD69 TNF CTLA4 TIGIT C1QA CCL3 HLA-DRA LYZ SFRP2 WISP2 TAGLN MYL9 POSTN COL3A1 RGS5 STEAP4 TM4SF1 IFI27 PTPRC SRSF7 XCL1 XCL2 LY6D KRT1 CD37 BANK1 KRT17 PTCH1 IL1B CXCL8 TPSB2 KIT PMEL ACAN COL2A1 CST3 CPVL CCL21 MMRN1 JCHAIN IGHA1 PTGDS ASPN CCL17 CCL22 FCER1A CDH19 SCN7A ACTA2 DES MYLPF
Percent Expressed
0
25
50
75
Average Expression
2
1
0
-1

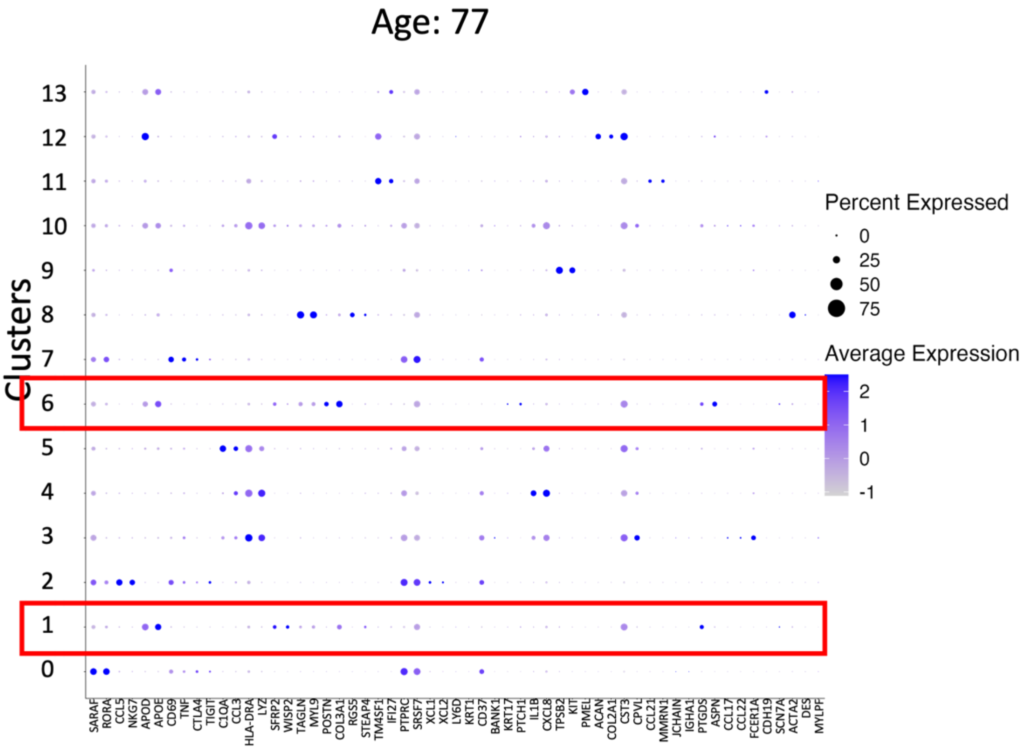
Age: 77
Clusters
13
12
11
10
9
8
7
6
5
4
3
2
1
0
SARAF RORA CCL5 NKG7 APOD APOE CD69 TNF CTLA4 TIGIT C1QA CCL3 HLA-DRA LYZ SFRP2 WISP2 TAGLN MYL9 POSTN COL3A1 RGS5 STEAP4 TM4SF1 IFI27 PTPRC SRSF7 XCL1 XCL2 LY6D KRT1 CD37 BANK1 KRT17 PTCH1 IL1B CXCL8 TPSB2 KIT PMEL ACAN COL2A1 CST3 CPVL CCL21 MMRN1 JCHAIN IGHA1 PTGDS ASPN CCL17 CCL22 FCER1A CDH19 SCN7A ACTA2 DES MYLPF
Percent Expressed
0
25
50
75
Average Expression
2
1
0
-1

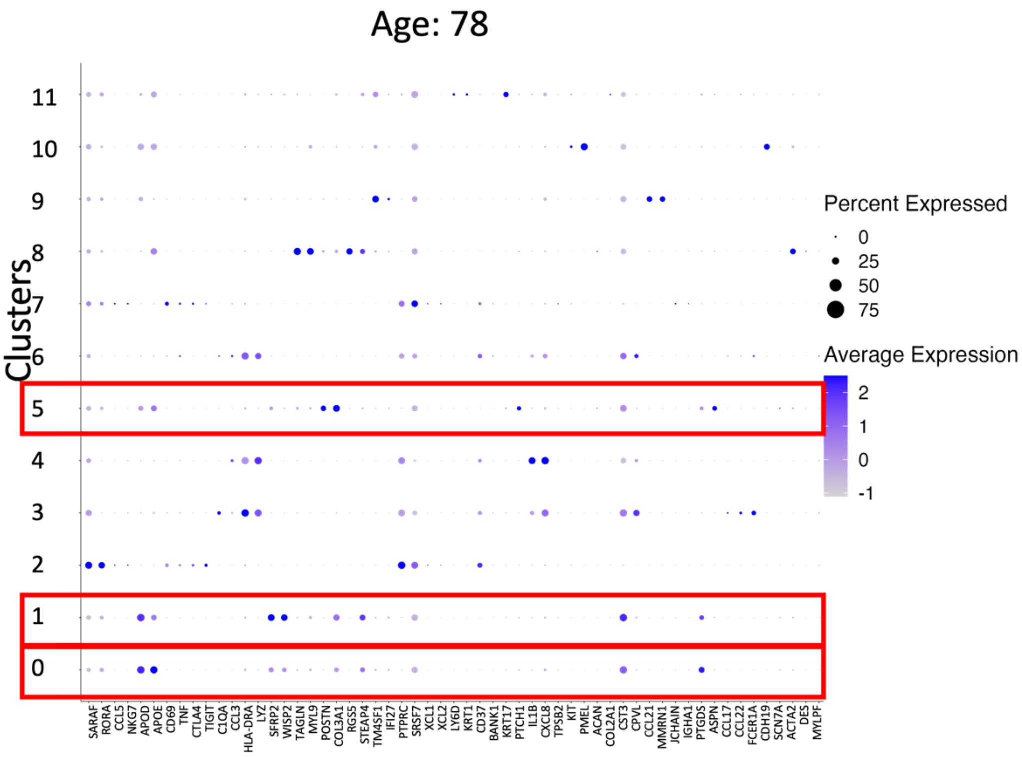
Age: 78
Clusters
11
10
9
8
7
6
5
4
3
2
1
0
Percent Expressed
0
25
50
75
Average Expression
2
1
0
-1
SARAF RORA CCL5 NKG7 APOD APOE CD69 TNF CTLA4 TIGIT C1QA CCL3 HLA-DRA LYZ SFRP2 WISP2 TAGLN MYL9 POSTN COL3A1 RGS5 STEAP4 TM4SF1 IFI27 PTPRC SRSF7 XCL1 XCL2 LY6D KRT1 CD37 BANK1 KRT17 PTCH1 IL1B CXCL8 TPSB2 KIT PMEL ACAN COL2A1 CST3 CPVL CCL21 MMRN1 JCHAIN IGHA1 PTGDS ASPN CCL17 CCL22 FCER1A CDH19 SCN7A ACTA2 DES MYLPF

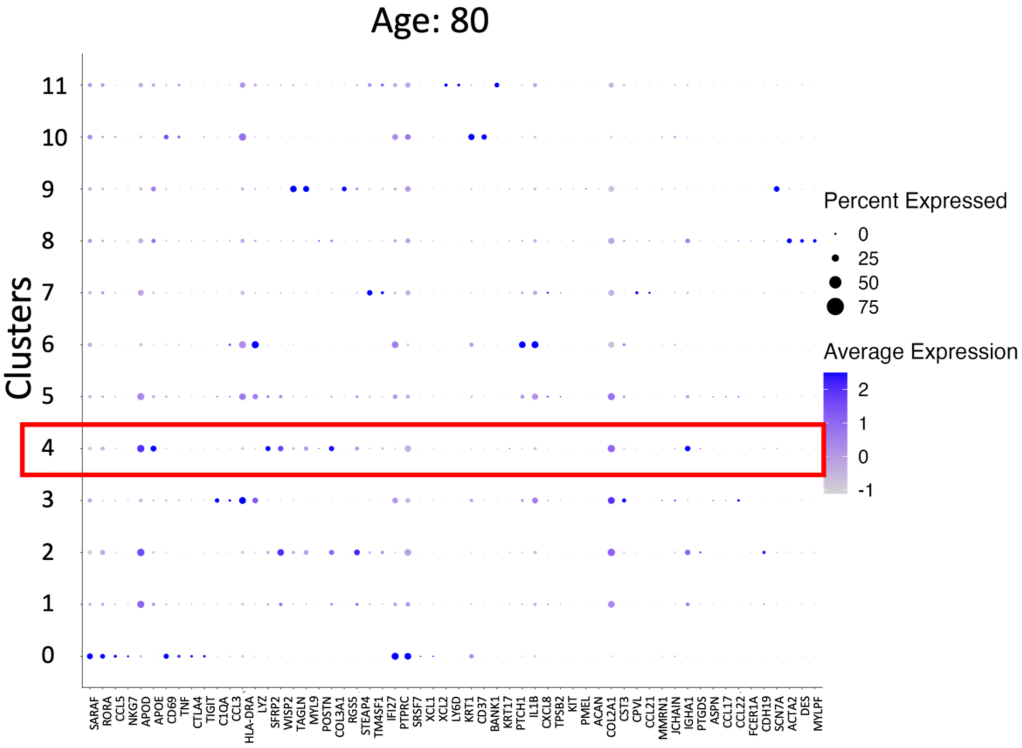
Age: 80
Clusters
11
10
9
8
7
6
5
4
3
2
1
0
Percent Expressed
0
25
50
75
Average Expression
2
1
0
-1
SARAF RORA CCL5 NKG7 APOD APOE CD69 TNF CTLA4 TIGIT C1QA CCL3 HLA-DRA LYZ SFRP2 WISP2 TAGLN MYL9 POSTN COL3A1 RGS5 STEAP4 TM4SF1 IFI27 PTPRC SRSF7 XCL1 XCL2 LY6D KRT1 CD37 BANK1 KRT17 PTCH1 IL1B CXCL8 TPSB2 KIT PMEL ACAN COL2A1 CST3 CPVL CCL21 MMRN1 JCHAIN IGHA1 PTGDS ASPN CCL17 CCL22 FCER1A CDH19 SCN7A ACTA2 DES MYLPF

37

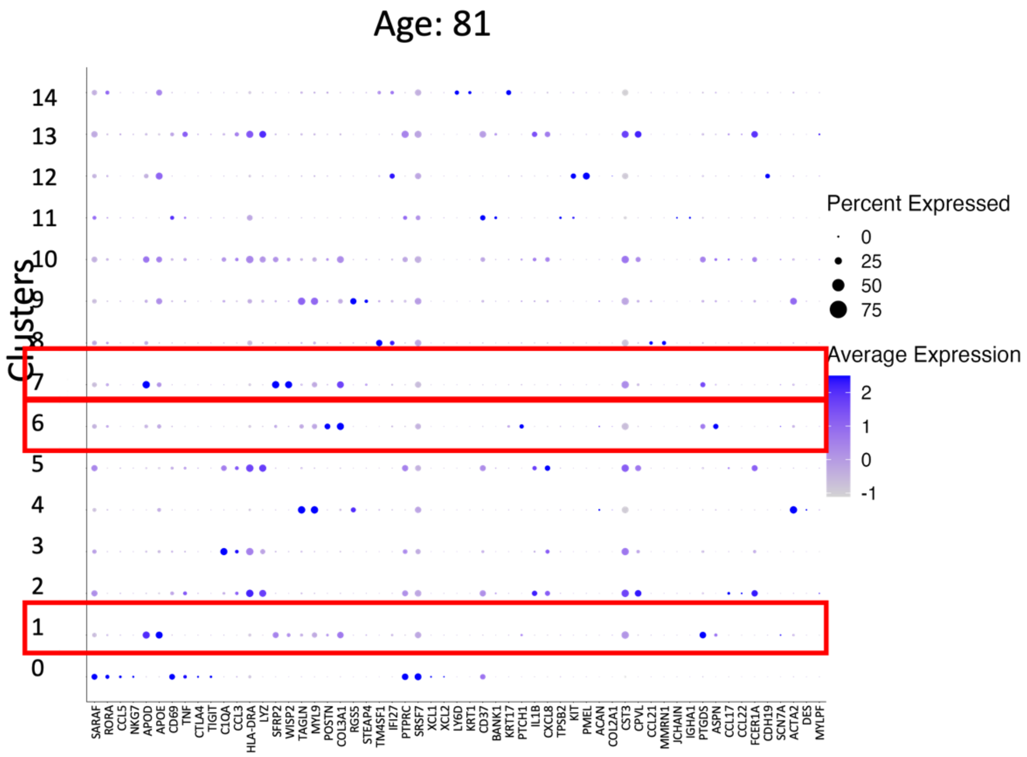
Age: 81
Clusters
14
13
12
11
10
9
8
7
6
5
4
3
2
1
0
SARAF RORA CCL5 NKG7 APOD APOE CD69 TNF CTLA4 TIGIT C1QA CCL3 HLA-DRA LYZ SFRP2 WISP2 TAGLN MYL9 POSTN COL3A1 RGS5 STEAP4 TM4SF1 IFI27 PTPRC SRSF7 XCL1 XCL2 LY6D KRT1 CD37 BANK1 KRT17 PTCH1 IL1B CXCL8 TPSB2 KIT PMEL ACAN COL2A1 CST3 CPVL CCL21 MMRN1 JCHAIN IGHA1 PTGDS ASPN CCL17 CCL22 FCER1A CDH19 SCN7A ACTA2 DES MYLPF
Percent Expressed
0
25
50
75
Average Expression
2
1
0
-1

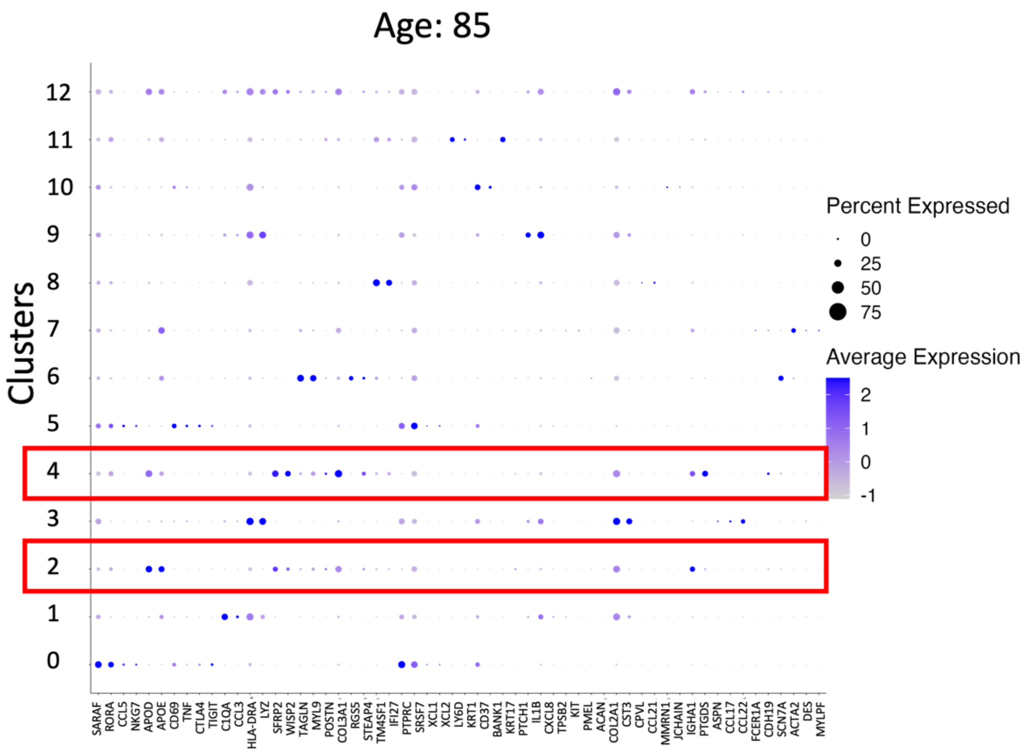
Age: 85
Clusters
12
11
10
9
8
7
6
5
4
3
2
1
0
SARAF RORA CCL5 NKG7 APOD APOE CD69 TNF CTLA4 TIGIT C1QA CCL3 HLA-DRA LYZ SFRP2 WISP2 TAGLN MYL9 POSTN COL3A1 RGS5 STEAP4 TM4SF1 IFI27 PTPRC SRSF7 XCL1 XCL2 LY6D KRT1 CD37 BANK1 KRT17 PTCH1 IL1B CXCL8 TPSB2 KIT PMEL ACAN COL2A1 CST3 CPVL CCL21 MMRN1 JCHAIN IGHA1 PTGDS ASPN CCL17 CCL22 FCER1A CDH19 SCN7A ACTA2 DES MYLPF
Percent Expressed
0
25
50
75
Average Expression
2
1
0
-1

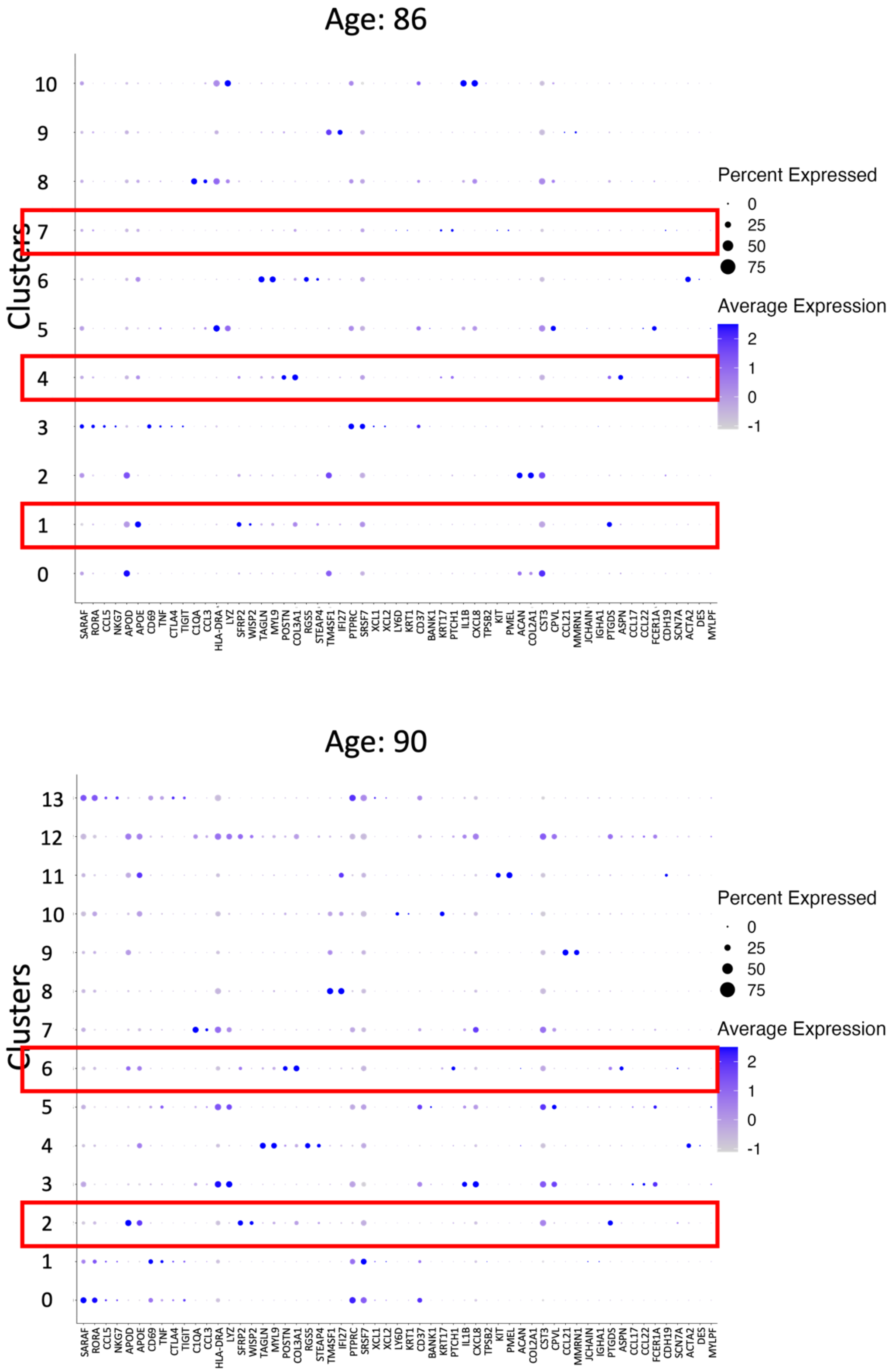

Fig S7. Dotplots of individual donor clusters from Ganier *et al.*[30] to identify and filter fibroblasts. Colour indicates mean normalised gene counts for each cluster, and the size of the dot indicates the percentage of cells within the cluster which express the gene.

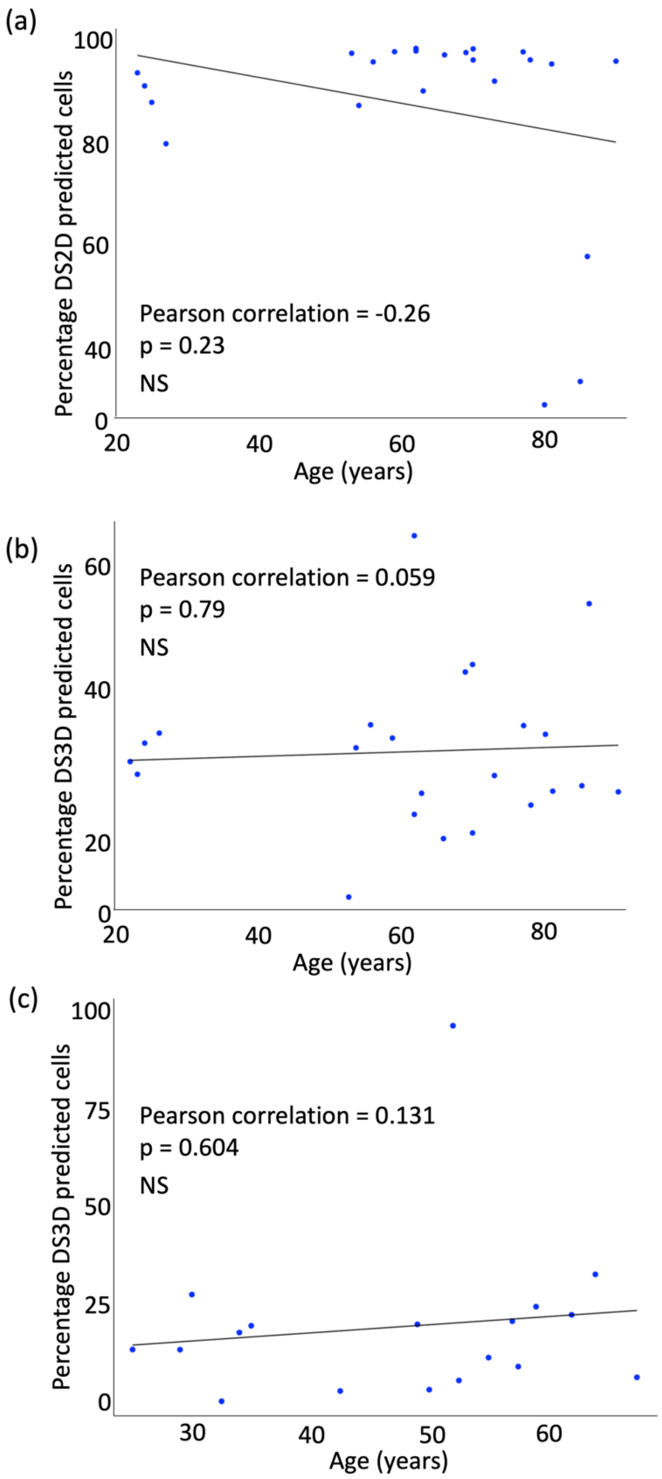

Fig S8. Scatterplots testing SenPred on dermal fibroblasts, whole skin, and lung fibroblasts. (a) Percentage of 2D SenPred senescent predicted dermal fibroblasts in Tabib[20], Solé-Boldo[21], and Ganier[30] datasets. (b) Percentage of 3D SenPred senescent predicted skin cells in Tabib[20], Solé-Boldo[21], and Ganier[30] datasets. (c) Percentage of 3D SenPred senescent predicted lung fibroblast cells from Sikkema *et al.*[31]

Table S1. Evaluation metrics for machine learning models built using 2D Early Proliferative (EP) and Deeply Senescent (DS) Human Dermal Fibroblasts (HDFs).

**Model**	**ROC**	**Sensitivity**	**Specificity**
Mixture Discriminant Analysis (MDA)	1	0.999	1
Support Vector Machine (SVM)	*1*	*0.999*	*0.99*
K-Nearest Neighbours (KNN)	0.998	0.978	0.992
Generalised Linear Model (GLM)	0.999	0.996	0.994
